# Phosphine Oxides as Spectroscopic Halogen Bond Descriptors: IR and NMR Correlations with Interatomic Distances and Complexation Energy

**DOI:** 10.3390/molecules25061406

**Published:** 2020-03-19

**Authors:** Alexei S. Ostras’, Daniil M. Ivanov, Alexander S. Novikov, Peter M. Tolstoy

**Affiliations:** Institute of Chemistry, St. Petersburg State University, 198504 St. Petersburg, Russia; st052055@student.spbu.ru (A.S.O.); dan15101992@gmail.com (D.M.I.); a.s.novikov@spbu.ru (A.S.N.)

**Keywords:** halogen bond, phosphine oxide, ^31^P NMR spectroscopy, IR spectroscopy, non-covalent interactions, spectral correlations

## Abstract

An extensive series of 128 halogen-bonded complexes formed by trimethylphosphine oxide and various F-, Cl-, Br-, I- and At-containing molecules, ranging in energy from 0 to 124 kJ/mol, is studied by DFT calculations in vacuum. The results reveal correlations between R–X⋅⋅⋅O=PMe_3_ halogen bond energy Δ*E*, X⋅⋅⋅O distance *r*, halogen’s σ-hole size, QTAIM parameters at halogen bond critical point and changes of spectroscopic parameters of phosphine oxide upon complexation, such as ^31^P NMR chemical shift, Δ*δ*P, and P=O stretching frequency, Δν. Some of the correlations are halogen-specific, i.e., different for F, Cl, Br, I and At, such as Δ*E*(*r*), while others are general, i.e., fulfilled for the whole set of complexes at once, such as Δ*E*(Δ*δ*P). The proposed correlations could be used to estimate the halogen bond properties in disordered media (liquids, solutions, polymers, glasses) from the corresponding NMR and IR spectra.

## 1. Introduction

Halogen bonding is one of the most abundant non-covalent interactions in chemistry [1,2]. Due to the anisotropic distribution of electron density around the covalently bound halogen atom, it has two distinct regions: (a) the region of increased electron density (nucleophilic site), located perpendicular to the covalent bond and corresponding to negative values of electrostatic potential (ESP) and (b) the region of decreased electron density (electrophilic site), also called σ-hole [3], located along the covalent bond. It is the existence of the electron-depleted σ-hole that determines the ability of the halogen atom to participate in attractive interactions with electron-donating atoms or groups [4,5,6], forming the so-called halogen bond R–X⋅⋅⋅Y (X—halogen atom). In halogen-bonded complexes, the X…Y distances are usually shorter than the sum of van der Waals radii of X and Y atoms, while the RXY angles tend to be close to 180°, because the σ-hole region is located on the continuation of the R–X axis. The range of halogen bond interaction energies is similar to that for hydrogen bonds, spanning from a fraction of kJ/mol up to 150 kJ/mol [7,8].

The formation of halogen bonds can be detected in solids [9,10,11,12,13], in liquids and solutions [14,15,16,17,18] and in gas phase [19,20,21]. Halogen bonding is being actively studied, and it has been demonstrated that it plays a significant role in biochemistry [22,23], in crystal design and design of functional materials (liquid crystals, molecular receptors, conductors, luminescence emitters, non-linear optical materials, etc.) [24,25,26,27,28,29,30,31,32], in organocatalysis [33,34,35,36,37,38] and in design of pharmaceuticals (here, halogen bonds are considered primarily as a type of hydrophobic functional groups, increasing the lipophilic properties of molecules and allowing them to pass through cell membranes) [39,40]. Besides, the halogen bond has been a subject of numerous theoretical works [41,42,43,44,45,46,47].

The main characteristics of halogen bond include (a) the geometric parameters (interatomic distances and angles, which could be estimated from diffraction studies) and (b) the complexation energy (experimentally available using, for example, calorimetric methods, though in condensed phases and for intramolecular interactions the definition of complexation energy might be ambiguous). In practice, it is often needed to characterize halogen bonds in disordered states of the matter, where in many cases it is done by indirect approaches, such as analysis of spectroscopic data based on previously established correlations. As a spectroscopic descriptor in such correlational methods, it is often convenient to take the change of a given parameter upon complexation, i.e., the difference between that parameter for the complex and the free molecule (which could be a probe molecule).

The main goal of this work is to propose a new spectroscopic method for the quantitative characterization of geometry and energy of halogen bonds. For this purpose we have selected trimethylphosphine oxide, Me_3_PO, as a model probe molecule. As spectroscopic descriptors of the halogen-bonded complexes formed by Me_3_PO, we have chosen (a) the change of the frequency of P=O stretching vibration upon complexation, Δν, and (b) the change of the ^31^P NMR chemical shift upon complexation, ΔδP. The choice of Me_3_PO as a probe molecule is stipulated by the fact that, due to the high polarization of the P=O bond, the terminal oxygen atom is an effective electron donor in non-covalent interactions, i.e., it is an effective halogen bond acceptor. For the same reason, it is expected that P=O coordination leads to substantial displacement of vibrational bands in IR spectra. Besides, the relatively rigid skeleton of Me_3_PO minimizes the influence on ΔδP of factors such as conformation of substituents (which becomes larges already for more flexible triethylphosphine oxide, let alone triphenylphosphine oxide).

Phosphine oxides in general are perspective probes for the diagnostics of non-covalent interactions. Intermolecular hydrogen-bonded complexes of phosphine oxides and their related compounds are discussed in several publications [48,49,50,51]. Moreover, there are several publications where the participation of phosphine oxides in halogen bonds is considered. For example, in [52,53], the crystal adducts of various phosphine oxides (Ph_2_(Me)P=O, Ph_3_PO) with strong halogen donors such as pentafluoroiodoebenzene C_6_F_5_I and 1,4-diaryl-5-iodotriazole are described. Finally, for a series of crystals containing molecules with P=O groups, the presence of short X∙∙∙O contacts (X = Cl, Br, I), which can be interpreted as halogen bonds, has been established with the help of X-ray analysis and quantum-chemical calculations [54,55,56,57,58,59,60,61]. Many other examples of XBs with phosphine oxides and related compounds can be found in CCDC data, which allowed us to perform an extensive database search and analyze the distributions of geometric parameters, as described in Section 4. The high electron-donating ability of phosphine oxides previously has been employed in the Gutmann–Beckett method to characterize the acceptor properties of solvents [62] and other compounds exhibiting Lewis acidity [63,64], expressed in so-called acceptor numbers, AN [65]. The main experimental parameter in this method is the change of the ^31^P NMR chemical shift of triethylphosphine oxide upon complexation, ΔδP, normalized to 100 for the complex with SbCl_5_.

In this work we have used the following criteria for the selection of halogen donor molecules: (i) relatively simple structure; (ii) presence of an electron-accepting group, which increases the positive ESP value in the σ-hole region and, consequently, enhances the electron-accepting ability of the halogen; (iii) absence of acidic hydrogen atoms that could compete with halogen bond by forming hydrogen bonds. Based on these criteria, we have selected an extensive set of 128 halogen-containing neutral molecules belonging to different classes of inorganic and organic chemical compounds R–X, where for simplicity RX stands also for R_3_X and R_5_X in case of halogen(III) and halogen(V) compounds, respectively:

(1) halogens (F_2_, Cl_2_, Br_2_, I_2_, At_2_);
(2) interhalides (ClF, ClF_3_, ClF_5_, BrF, BrF_3_, BrF_5_, BrCl, IF, IF_3_, IF_5_, ICl, ICl_3_, IBr, AtCl, AtBr, AtI);
(3) oxohalides (OF_2_, ClO_3_OF, Cl_2_O, ClO_2_, ClO_2_F, ClO_3_OCl, Br_2_O, BrO_2_, BrO_2_F, ClO_3_OBr, IO_2_F, ClO_3_OI);
(4) pseudohalides (FCN, FN_3_, FCNO, ClCN, ClN_3_, ClNCO, ClSCN, BrCN, BrN_3_, BrNCO, BrSCN, ICN, IN_3_, INCO, ISCN);
(5) halogenated methanes and their derivatives (CF_3_OF, CF_3_SO_2_OF, CF_3_Cl, CCl_2_F_2_, CCl_3_F, CCl_4_, CF_3_OCl, CF_3_SO_2_OCl, CF_3_Br, CBr_2_F_2_, CBr_3_F, CBrCl_3_, CBrClF_2_, CBr_4_, CF_3_OBr, CF_3_SO_2_OBr, CF_3_I, CI_2_F_2_, CI_3_F, CIClF_2_, CI_4_);
(6) halogenated ethylene, halogenated acetylene and their derivatives (C_2_F_4_, C_2_Cl_4_, C_2_F_3_Cl, C_2_Br_4_, C_2_F_3_Br, C_2_I_4_, C_2_F_3_I, C_2_(CN)_3_Cl, C_2_F_2_, C_2_Cl_2_);
(7) phosgene and its derivatives (COF_2_, COClF, COCl_2_, COBrCl, COBr_2_, COBrF, COIF);
(8) thionyl- and sulfurylhalides (SOF_2_, SO_2_ClF, SOCl_2_, SOBr_2_, SO_2_Cl_2_, SO_2_BrF);
(9) sulfur halides and sulfur hypohalites (SF_6_, SF_5_OF, SF_5_Cl, SF_5_OCl, S_2_Cl_2_, SCl_2_, SF_5_Br, S_2_Br_2_, SBr_2_);
(10) halogenated nitrogen-containing inorganic compounds (NF_3_, NOF, NO_2_F, NO_2_OF, NCl_3_, NF_2_Cl, NOCl, NO_2_Cl, NO_2_OCl, NBr_3_, NF_2_Br, NOBr, NO_2_Br, NO_2_OBr, NI_3_);
(11) assorted organic compounds (tetrafluoro-1,4-benzoquinone, tetrachloro-1,4-benzoquinone, tetrabromo-1,4-benzoquinone, tetraiodo-1,4-benzoquinone, C_6_H_5_(C≡C)Cl, C_6_H_5_(C≡C)Br, C_6_H_5_(C≡C)I, CCl(CN)_3_, CBr(CN)_3_, N-chlorosuccinimide, N-bromosuccinimide, N-iodosuccinimide).

**Scheme 1:**
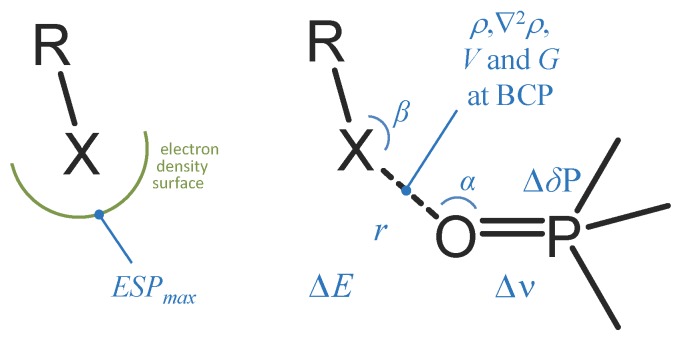
Schematic structure of 1:1 halogen-bonded complexes formed by Me_3_PO with RX (X—halogen) and the list of calculated parameters studied in this work: *r—*X⋅⋅⋅O distance; *α* and *β—*X⋅⋅⋅O=P and R–X⋅⋅⋅O angles, respectively; ΔE—complexation energy (BSSE corrected); *ρ*, ∇^2^*ρ*, *V* and *G* are electron density, Laplacian of electron density, local electron potential and kinetic energies densities at halogen bond critical point (3; –1), respectively; *ρ* and ∇^2^*ρ* are electron density and Laplacian of electron density; Δ*δ*P—change of ^31^P NMR chemical shift upon complexation; Δν –change of harmonic P=O stretching wavenumber upon complexation; *ESP*_max_*—*the extremal value of electrostatic potential in the region of σ-hole on the surface of equal electron density taken at 0.001 electron/Bohr^3^ level.

For each 1:1 complex formed by Me_3_PO molecule and one of the abovementioned halogen donors, we have quantum-chemically calculated (M06-2X/def2-TZVPPD level of theory [66]) the equilibrium geometry, the harmonic vibrational frequencies, the nuclear chemical shielding constants, the complexation energies (corrected for BSSE, the basis set superposition error) and the value of ESP in the σ-hole region for the free halogen donor molecule. Besides, we have calculated and analyzed the electron density properties at the (3; –1) halogen bond critical point (BCP) using the Bader’s quantum theory of atoms in molecules (QTAIM) methodology [67]. The parameters which are determined and analyzed in this work are shown in Scheme 1. 

## 2. Results and Discussion

The calculated geometries of all complexes are shown in Figure S1, and all the parameters considered in this work (see Scheme 1) are collected in Tables S1 and S2. In this section we will present only the plots and fitting equations that are necessary for the discussion.

### 2.1. Angular Distribution

**Figure 1:**
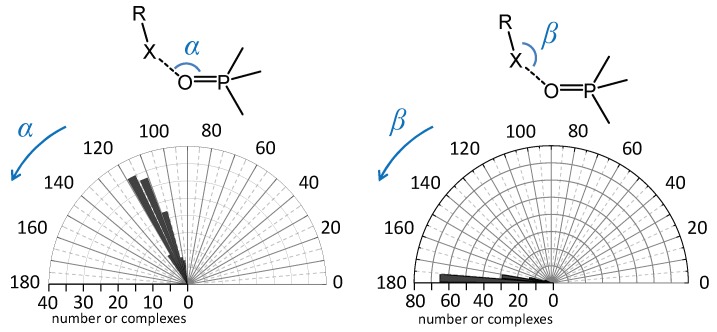
Distribution of angles *α* (X⋅⋅⋅O=P) and *β* (R–X⋅⋅⋅O) for 128 complexes studied in this work (X = F, Cl, Br, I, At). Histogram bars are taken every 10° for *α* and every 5° for *β*.

Based on the calculated optimized geometries of halogen-bonded R–X⋅⋅⋅O=P complexes (X = F, Cl, Br, I, At), the distributions of angles *α* (angle X⋅⋅⋅O=P) and *β* (angle R–X⋅⋅⋅O) were constructed. In case of multivalent halogen bond donors, such as some of interhalides, the angle *β* was chosen as the largest one among all possible R–X⋅⋅⋅O values. In Figure 1, the resulting distributions are shown as histograms in which the bar height is proportional to the number of complexes having the corresponding angle within the given 5° range.

The values of angle *α* lie primarily in the range 100–120°. This indicates that the halogen bonds are expectedly formed along the direction of oxygen lone pairs. For the *sp*^2^-hybridized oxygen, the angle between the P=O bond and the oxygen lone pair should be 120°, though the high polarization of the PO bond increases the contribution of the P^+^–O^−^ resonance structure, corresponding to the *sp*^3^ hybridization, which makes *α* closer to the tetrahedral angle 109.5°. Thus, the distribution of angles *α* might be considered as an indication that the phosphine oxide structure is intermediate between the neutral one, P=O, and the zwitterionic one, P^+^–O^−^. Further deviations of angles α are most probably due to the presence of secondary interactions or steric hindrance.

The angle *β* describes how linear the halogen bond is. The values of *β* are influenced by the shape and size of the σ-hole on the halogen atom. As σ-holes are located along the continuation of the R–X bond, the values of *β* lie close to 180°. Overall, the stronger and shorter the halogen bond is, the more linear it is (see Figure S2). It stands to reason that the complexation energy increases with the increase of the σ-hole, which in turn depends on the polarizability of the halogen atom; thus, it could be expected that, in general, the broader range of angles *β* would correspond to the weakest R–F⋅⋅⋅O=P complexes, and the narrow range of *β* values close to 180° would be observed for stronger R–I⋅⋅⋅O=P and R–At⋅⋅⋅O=P complexes. Indeed, this is the case within the series of complexes considered in this work (see Figure S2).

**Figure 2:**
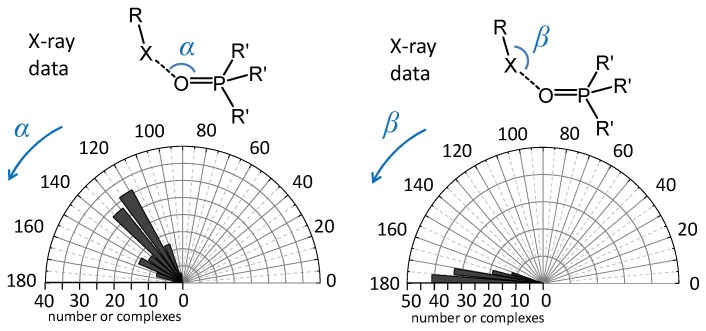
Distribution of angles *α* (X⋅⋅⋅O=P) and *β* (R–X⋅⋅⋅O) for CCDC 2020 structures containing R–X⋅⋅⋅O=P fragment (X = Cl, Br, I). The see complete list of entries in Table S3. Histogram bars are taken every 10° for *α* and every 5° for *β*.

The abovementioned angular distributions could be compared with the results of the statistical analysis of CCDC 2020 data for phosphine oxides and related PO-containing compounds as O-nucleophiles for halogen bond. Note that only three halogens (Cl, Br, I) were considered, because (i) no results for At were found due to all astatine isotopes being radioactive; and (ii) although we found two structures (XAQYOF and XUFQOF) for fluorine, this information is definitely not enough to collect statistics. The values of angle *α* for this statistical data lie primarily in the range 120–140°, with a median value of 129.4°, as shown in Figure 2. This indicates that the halogen bonds are still preferably formed along the direction of oxygen lone pairs, but, in case of bulk substituents around P=O functionality (larger than CH_3_ groups in the model trimethylphosphine oxide), the halogen donors have to occupy positions with larger values of *α*, which is less optimal for the halogen bond stabilization; even almost linear complexes are possible (the largest *α* value is 173.2° in OBUCAQ).

**Figure 3:**
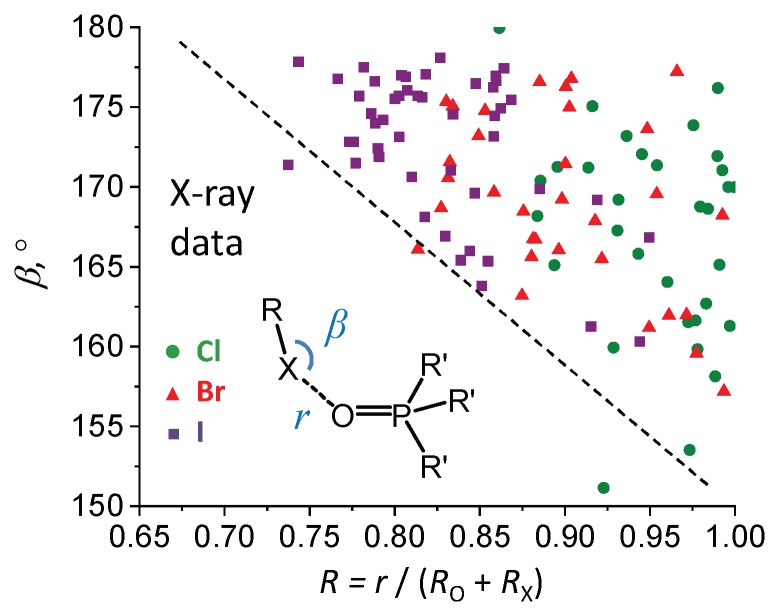
Correlation between angles *β* (R–X⋅⋅⋅O) and normalized distance parameter *R* = *r*/(*R*_O_ + *R*_X_), where *r* is X⋅⋅⋅O distance and *R*_O_ and *R*_X_ are van der Waals radii of oxygen and halogen atoms, respectively, for CCDC 2020 structures containing R–X⋅⋅⋅O=P fragment (X = Cl, Br, I). For the complete list of entries see Table S3.

The R–X⋅⋅⋅O fragments are less strictly linear for the CCDC data than for theoretically investigated R–X⋅⋅⋅OPMe_3_ models (see Figure 2). However, the median value of angle *β* is 170.1°, which is still rather close to 180°. As seen from the calculated data, the stronger and shorter the halogen bond is, the more linear it is (see Figure 3). Note that for all halogens under consideration (Cl, Br, I) combined and for each halogen individually it is possible to draw the one cut-off line in Figure 3 above which almost all data points will be located.

### 2.2. Complexation Energy Dependence On Intermolecular Distance

There seems to be no universal dependence which can be used to estimate the halogen bond energy using the interatomic distances and bond angles. The previously reported correlations were tested on relatively homologous sets of intermolecular complexes [47,68]. Here, using our set of 128 complexes with participation of various halogens, we attempt to construct energy–structure correlations which on one hand would be general enough (i.e., would have similar form for all halogen donor types) and on the other hand would be simple enough, allowing for a rapid energy estimation.

**Figure 4:**
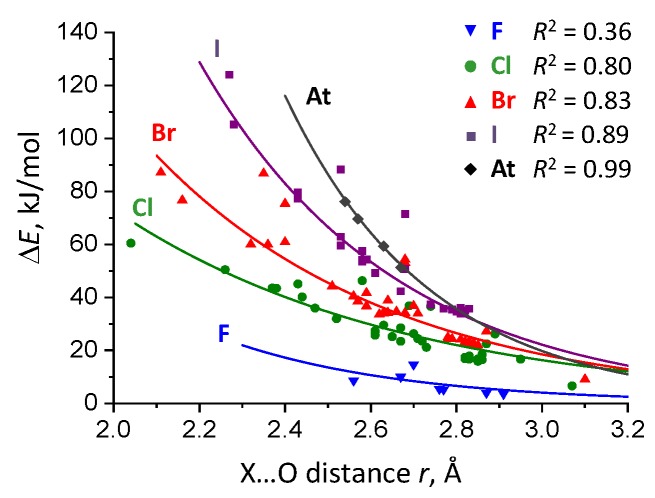
Correlation between calculated complexation energy Δ*E* and interatomic distance *r* (X⋅⋅⋅O, where X = F, Cl, Br, I, At) for R–X⋅⋅⋅O=PMe_3_ halogen-bonded complexes studied in this work. The solid curves correspond to Equation (1).

Figure 4 shows the correlations between calculated complexation energies Δ*E* and X⋅⋅⋅O interatomic distances for all complexes studied in this work. The majority of data points in the plot are located in the range of X⋅⋅⋅O distances from 2.4 to 3.1 Å, while the overall span of complexation energies is from 3.6 up to 124 kJ/mol.

Quite expectedly, for each given halogen (F, Cl, Br, I or At) the decrease of the X⋅⋅⋅O distance *r* is accompanied by the increase of complexation energy Δ*E*. The shape of this correlation could be approximated by exponentially decaying functions such as:

(1) $$∆E\left(\mathrm{kJ}/\mathrm{mol}\right)=A\cdot e^{-b\cdot r},$$

where *A* (in kJ/mol) and *b* (in Å^−1^) are fitting coefficients, individual for each halogen. The corresponding fitted curves are added to Figure 4 as solid lines, while the numerical values of parameters *A* and *b* are collected in Table 1. One can see that the fitted parameters found for complexes with fluorine-containing molecules are falling out of the overall trend, which is probably associated not only with the small number of data points, but also with the weak ability of fluorine to act as an electron acceptor (due to the small—sometimes almost absent—σ-hole).

It should be noted that the quality of the correlation increases in the order from chlorine- to astatine-containing halogen donors (see the corresponding R^2^ values added to Figure 2), because for heavier atoms with larger σ-hole (Br, I, At) the halogen bond gets generally stronger and it becomes the dominant factor determining the interatomic distance X⋅⋅⋅O.

**Figure 5:**
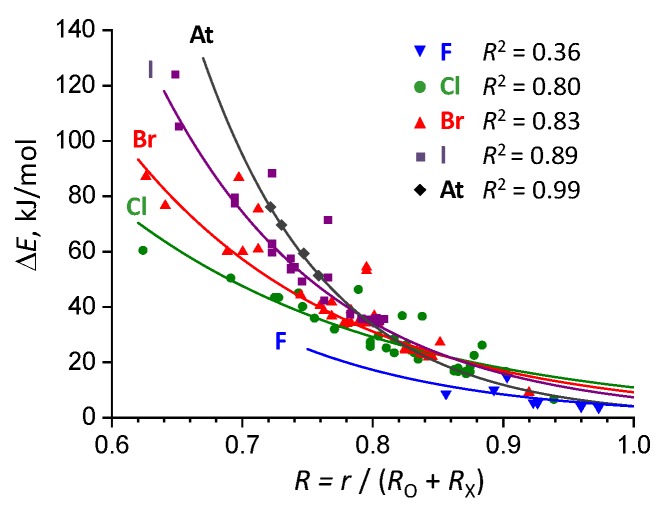
Correlation between calculated complexation energy Δ*E* and normalized distance parameter *R* = *r*/(*R*_O_ + *R*_X_), where *r* is X⋅⋅⋅O distance and *R*_O_ and *R*_X_ are van der Waals radii of oxygen and halogen atoms, respectively, for R–X⋅⋅⋅O=PMe_3_ halogen-bonded complexes studied in this work (X = F, Cl, Br, I, At). The solid curves correspond to Equation (3).

Partially, the differences between fitted parameters in Equation (1) for different halogens are explained by the fact that halogens simply increase in size going from F to At. Thus it is interesting to replot Figure 4, using for abscissa not the absolute values or *r* but the “reduced” values, normalized to the sum of van der Waals radii of oxygen and halogen:

(2) $$R=\frac{r}{R_{O}+R_{X}},$$

where *R*_O_ and *R*_X_ are van der Waals radii of oxygen and halogen, respectively. For this purpose we have used Bondi’s radii [69] (in Å: *R*_O_ = 1.52, *R*_F_ = 1.47, *R*_Cl_ = 1.75, *R*_Br_ = 1.85, *R*_I_ = 1.98, *R*_At_ = 2.02, the latter value taken from [70]). The result is shown in Figure 5. The *R* values could be considered as a measure of an overlap of atomic electron shells upon complexation: the closer *R* values are to unity, the weaker the halogen bond is. For example, as fluorine-containing molecules are weaker halogen donors in the series, the *R* values for their complexes are the largest; in contrast, for astatine-containing molecules, the *R* values are significantly smaller.

Due to the fact that *R* is a simple normalization of initial *r* values, the data sets in Figure 5 could be fitted by the same type of functions, as in Equation (1), with the same quality of the fit:

(3) $$∆E\left(\mathrm{kJ}/\mathrm{mol}\right)=A\cdot e^{-B\cdot R},$$

where the values of unitless parameters *B* are as listed in Table 1. The spread of fitting curves in Figure 5 is significantly smaller than that in Figure 4, though there is still some difference in behavior of different halogens. Due to the increase of polarizability the decrease of electronegativity and, therefore, increase of maximal *σ*-hole potential of halogens from F to At, the shortening of the X⋅⋅⋅O bond leads to a more effective increase of the interaction energy for At and I, as compared to other halogens. This effect is still noticeable even if *R* values (Figure 5) are taken instead of *r* values (Figure 4) for the correlation.

The proposed correlational functions could be used to estimate the halogen bond energy in crystals in case the interatomic distance is known. Considering the scattering of data points, the precision of such estimation would be roughly ±20% over the whole range of energies. The applicability of the correlations is probably limited to halogen-bonded complexes of X⋅⋅⋅O type; for X∙∙∙N, X∙∙∙S, X∙∙∙P and X∙∙∙X interactions the fitted parameters might differ. Nevertheless, it could be expected that qualitatively the type fitting function would remain unchanged.

### 2.3. Complexation Energy Dependence On σ-hole Electrostatic Characteristics

**Figure 6:**
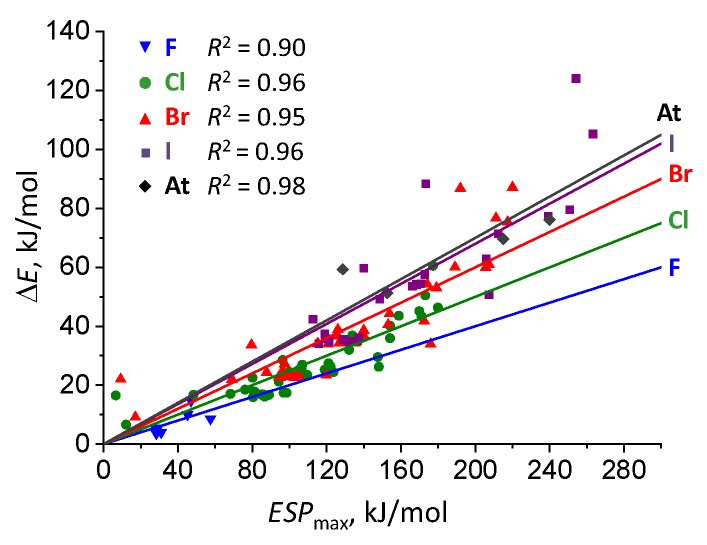
Correlation between calculated complexation energy Δ*E* and the extremal value of electrostatic potential *ESP*_max_, measured in the region of σ-hole of X atom on the surface of equal electron density taken at 0.001 electron/Bohr^3^ level for R–X⋅⋅⋅O=PMe_3_ halogen-bonded complexes studied in this work (X = F, Cl, Br, I, At). The solid lines correspond to Equation (4).

The halogen’s σ-hole could be characterized by *ESP*_max_, which is the maximal value of electrostatic potential measured on the surface of equal electron density. Usually for this purpose the electron density level of 0.001 electron/Bohr^3^ is selected. It is convenient to represent *ESP*_max_ values in the units of energy, kJ/mol, as the energy of interaction with a virtual unit probe charge. The *ESP*_max_ values for the halogen donor molecules considered in this work are listed in Table S1 and plotted versus Δ*E* in Figure 6. It is possible to fit the data sets with linear equations:

(4) $$∆E\left(\mathrm{kJ}/\mathrm{mol}\right)=D\cdot ESP_{max},$$

where the unitless parameters *D* are again individual for a given halogen. The numerical values of coefficients *D* are collected in Table 1. The correlation lines (see Figure 6) pass through the origin, which means that the halogen bond does not form in the absence of σ-hole (Δ*E* = 0). Some of the deviations of the data points from linear correlations are possibly due to the presence of secondary non-covalent interactions, other than halogen bonding, i.e., not associated with the σ-hole on the halogen atom, such as various electrostatic and dispersion interactions.

### 2.4. Correlation Between Complexation Energy And P=O Stretching Frequency

**Figure 7:**
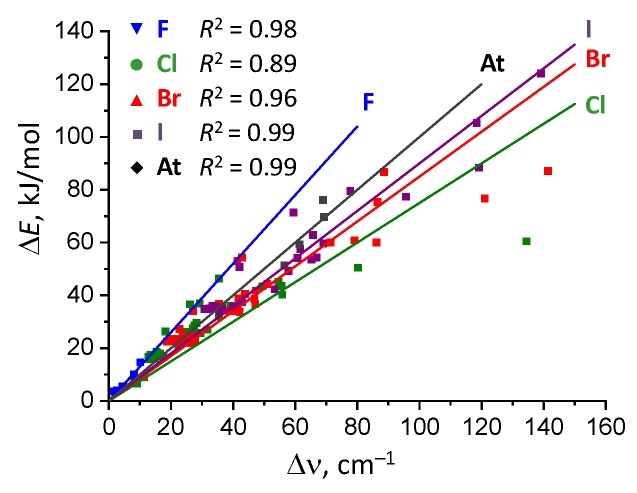
Correlation between calculated complexation energy Δ*E* and absolute value of the change of the P=O stretching vibration frequency upon complexation, Δν, for R–X⋅⋅⋅O=PMe_3_ halogen-bonded complexes studied in this work (X = F, Cl, Br, I, At). The solid lines correspond to Equation (5).

Vibrational spectroscopy is one the most widely used methods for identification and characterization of non-covalent interactions. The formation of an intermolecular complex is usually accompanied by the decrease of the stretching vibrational frequency of the electron-donating group. This frequency shift could be used to construct correlations, allowing one to indirectly estimate other parameters of the complex, such as its energy or geometry. In Figure 7 we show the Δ*E* dependence on the calculated P=O stretching vibration frequency shift upon complexation, Δν. The overall trend is apparent: the stronger is the complex, the larger is the frequency shift. This could be rationalized as follows: the formation of halogen-bonded complex leads to the electron density transfer on the σ* molecular orbital of the electron-accepting molecule. This in turn leads to the weakening and lengthening of the P=O bond, which results in the lowering of the harmonic force constant for the P=O stretching vibration.

The data sets shown in Figure 7 could be approximated by linear functions, passing through the origin:

(5) ∆E(kJ/mol)=K·Δν,

where *K* is the proportionality coefficient expressed in kJ/mol/cm^−1^ units. The numerical values of coefficients *K*, individual for each halogen, are collected in Table 1. As was the case for other correlations presented above, the data set for fluorine-containing molecules is subject to the largest approximation errors and is likely to perform poorly beyond the complexation energy of ca. 15 kJ/mol. In order to further simplify Equation (5)—admittedly with some loss of its accuracy—it is possible to propose one universal “average” equation, suited for rough energy estimations:

(6) ∆E(kJ/mol)=0.85·Δν,

where Δν is measured in cm^−1^. Here, as the average coefficient, we take the coefficient obtained by a linear fitting of the entire data set shown in Figure 7.

### 2.5. Correlation Between Complexation Energy And ^31^P NMR Chemical Shift

**Figure 8:**
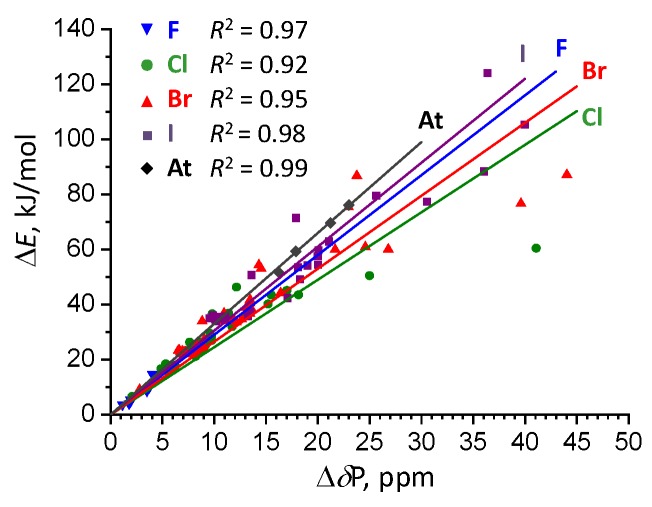
Correlation between calculated complexation energy Δ*E* and change of the ^31^P NMR chemical shift upon complexation, Δ*δ*P, for R–X⋅⋅⋅O=PMe_3_ halogen-bonded complexes studied in this work (X = F, Cl, Br, I, At). The solid lines correspond to Equation (7).

The ^31^P NMR chemical shift is very sensitive to the chemical structure of the phosphorous-containing compounds and to their environment. The “rule of thumb” is that the ^31^P NMR signal shifts to the high field due to the presence of electron-donating substituents and to the low field due to electron-accepting ones. This is consistent with what we observe in calculations: formation of halogen bond increases the ^31^P NMR chemical shift of Me_3_PO. This is illustrated in Figure 8, where a correlation between Δ*E* and Δ*δ*P is shown: the stronger the halogen bond is, the larger the signal shift to the low field is. The data sets plotted in Figure 6 can be fitted by linear functions:

(7) ∆E(kJ/mol)=M·ΔδP,

where coefficients *M* (in kJ/mol/ppm) slightly differ for F, Cl, Br, I and At. The numerical values are collected in Table 1. In a similar way as it was done for Δν, for Δ*δ*P it is possible to construct one universal correlation for rough estimations of complexation energies based of ^31^P NMR spectra:

(8) ∆E(kJ/mol)=2.7·ΔδP,

where Δ*δ*P is measured in ppm. Again, the average coefficient refers to the fitting of the entire data set shown in Figure 8. It should be noted that despite the generally high sensitivity of *δ*P to the molecular structure and to non-covalent interactions [71], in the literature there are only few attempts to use *δ*P for the solution of the reverse spectroscopic problem for non-covalent complexes, i.e., for the finding of the complex’s energy and structure based on the phosphorous chemical shift value [72,73,74,75]. Partially the reason for this might be in the high sensitivity itself, because contributions to *δ*P from various weak secondary non-covalent interactions might “smudge” the effect of the halogen bonding, thus strongly reducing the diagnostic value of the spectroscopic marker. In case of phosphine oxides R_3_PO, we hope that such probe molecules are reasonably rigid and less prone to secondary interactions than, for example, phosphinic acids; if so, the correlation proposed in this work would be of practical value.

**Figure 9:**
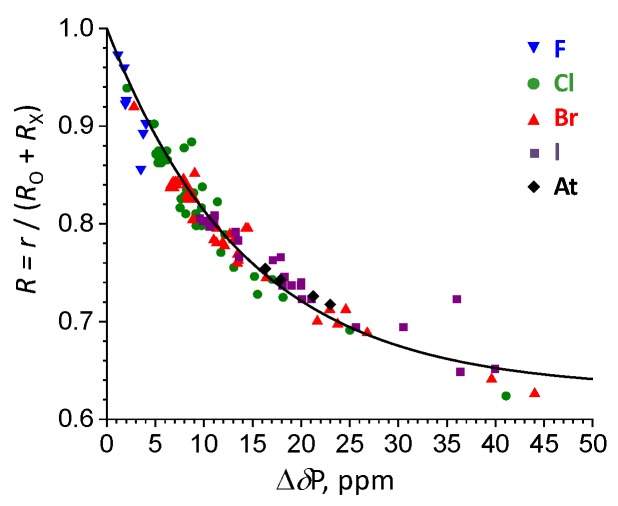
Correlation between the normalized distance parameter *R* = *r*/(*R*_O_ + *R*_X_), where *r* is X⋅⋅⋅O distance and *R*_O_ and *R*_X_ are van der Waals radii of oxygen and halogen atoms, respectively, and the ^31^P NMR chemical shift upon complexation, Δ*δ*P, for R–X⋅⋅⋅O=PMe_3_ halogen-bonded complexes studied in this work (X = F, Cl, Br, I, At). The solid line corresponds to Equation (9). *R*^2^ (coefficient of determination) of the fitted curve equals to 0.933.

In Figure S7 we test the possibility of using Δ*δ*P to estimate halogen atom’s electrophilicity, measured as *ESP*_max_ value. There is significant scattering of data points which has prevented us from proposing a correlation, though it could be speculated that the overall dependence is independent of the type of halogen. A much better universal correlation could be proposed between the reduced interatomic distance *R* (see Equation (2) for the definition) and Δ*δ*P (see Figure 9).

The data points in Figure 9 could be fit reasonably well with a single exponential function:

(9) $$R=0.63+0.37\cdot e^{-0.07\cdot \Delta \delta P},$$

where Δ*δ*P is measured in ppm.

**Figure 10:**
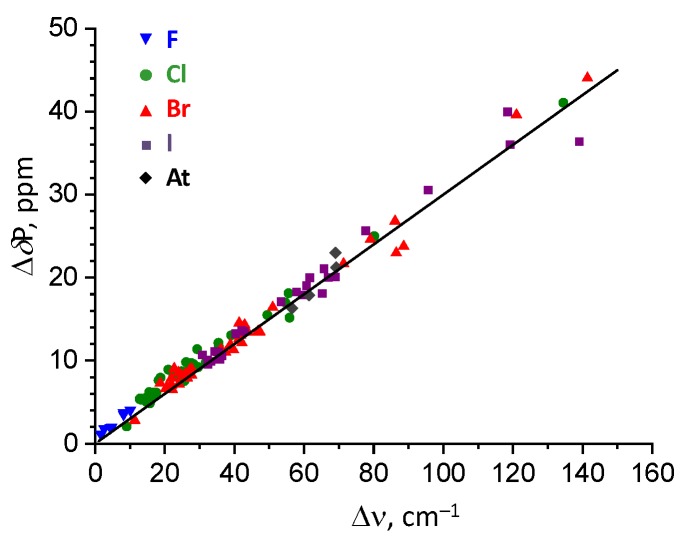
Correlation between the change of the P=O stretching vibration frequency upon complexation, Δν, and the change of the ^31^P NMR chemical shift upon complexation, Δ*δ*P, for R–X⋅⋅⋅O=PMe_3_ halogen-bonded complexes studied in this work (X = F, Cl, Br, I, At). The solid line corresponds to Equation (10). *R*^2^ (coefficient of determination) of the fitted line equals to 0.993.

From the dependencies shown in Figure 7 and Figure 10, one could expect that the effects of complexation on IR and NMR spectra are correlated. This is indeed the case, as shown in Figure 10; for all types of halogens, the data points could be approximated with a single linear dependence:

(10) ΔδP=0.3·Δν,

where Δ*δ*P is in ppm and Δν is in cm^−1^. The high degree of correlation between two independent spectral characteristics could serve as an indication of the robustness of the selected probe molecule (Me_3_PO in our case), as well as an indication that both in IR and NMR spectra the main effects (the change of the P=O stretching frequency and the change of the ^31^P NMR chemical shift) are caused by the donation of the electron density from one of the oxygen lone pairs to the halogen donor molecule.

### 2.6. QTAIM Analysis of the Electronic Structure of Complexes

Figures S3–S6 show the dependencies of complexation energy Δ*E* on QTAIM parameters at BCP, namely *G*, *V*, *ρ* and ∇^2^*ρ* (see Section 2 for more information). In this section, we summarize only the main observations. In all cases, the scattering of data points in plots is substantial, and no universal trends applicable for all halogens could be noticed. Nevertheless, it is possible to construct linear approximations such as:

(11) $$∆E\left(\mathrm{kJ}/\mathrm{mol}\right)=C_{G}\cdot G_{,}\;∆E\left(\mathrm{kJ}/\mathrm{mol}\right)=C_{V}\cdot V_{,}\;∆E\left(\mathrm{kJ}/\mathrm{mol}\right)=C_{rho}\cdot \rho_{,}\;∆E\left(\mathrm{kJ}/\mathrm{mol}\right)=C_{Lap}\cdot \nabla^{2}\rho_{,}$$

where independent variables are expressed in the following units: *G* in kJ/mol/Å^3^, *V* in kJ/mol/Å^3^ (both *V* and *G* were taken as absolute values), *ρ* in a.u./Å^3^ and ∇^2^*ρ* in a.u./Å^5^. The resulting fitting coefficients are collected in Table 2. In all cases, the quality of the fit (R^2^) was 0.8–0.9 for F-containing halogen bonded complexes and 0.91–0.99 for Cl^−^, Br^−^, I^−^ or At-containing ones. The proportionality coefficients in Equation (9) are not universal: they vary for different halogens; usage of one average coefficient would lead to significant errors in energy estimations. This observation is similar to the one made in [76], where similar coefficients were proposed for Cl-, Br- and I-containing halogen bonds (see the values listed in Table 2).

## 3. Materials and Methods

### 3.1. Computational Details

The full geometry optimization of all model structures in vacuum, complexation energies (including the relaxation energy of optimized monomers and corrected for the basis set superposition error by counterpoise method [77] as a single point calculation after the geometry optimization), ESP values and spectroscopic characteristics were calculated at the DFT level of theory using Gaussian 16 software (Gaussian Inc. Wallingford, CT, USA) [78]. The visualization was done using GaussView 6.0 (Gaussian Inc. Wallingford, CT, USA) [79] and ChemCraft [80] software.

For all calculations, we used the hybrid functional M06-2x, which was previously shown to perform well for the investigation of non-covalent interaction of small molecules [81,82,83,84,85]. Due to the correction for dispersion interactions, this functional is well suited for the estimation of geometry and energy of halogen bonds [86]. The basis set def2-TZVPPD was selected because it includes (i) polarization functions allowing for a better description of asymmetric electron distributions in halogens; (ii) diffuse functions which describe well the relatively long-distance non-covalent bonds and (iii) parametrization of pseudopotentials necessary to describe the relativistic effects for heavy halogens, such as I and At [87].

The calculations of harmonic vibrational frequencies were used to define the shift of the P=O stretching band upon complexation as Δν = ν_0_ − ν, where ν_0_ are ν are vibrational frequencies for the free Me_3_PO and Me_3_PO in complex, respectively. For the free Me_3_PO, ν_0_ = 1268 cm^−1^. Taking into account that for all studied complexes ν < ν_0_, the definition used for Δν makes its values positive, which is done for convenience. The calculations of chemical shielding were performed using GIAO method. The change of ^31^P NMR chemical shift upon complexation was defined as Δ*δ*P = σ_0_(^31^P) − σ(^31^P), where σ_0_(^31^P) and σ(^31^P) are shielding constants of ^31^P nucleus in the free Me_3_PO and Me_3_PO in complex, respectively. For the free Me_3_PO, σ_0_(^31^P) = 268 ppm.

The topological analysis of electron density at halogen bond critical points (BCPs) was carried out within the framework of QTAIM methodology using MultiWFN software [88]. The following BCP parameters were considered: local electron kinetic (*G*) and potential (*V*) energy densities, electron density *ρ* as well as its Laplacian ∇^2^*ρ*.

All of the abovementioned calculated halogen bond characteristics were correlated with complexation energy and in some cases between each other; the proposed linear and non-linear correlation functions were fitted by least squares method using Origin software [89]. The complexes in which the dominant interaction was not the R–X⋅⋅⋅O=P halogen bond (e.g., pnictogen bond, chalcogen bond or π-hole interaction) were not included into the regression analysis. Such complexes are marked with color in Figure S1 and Tables S1 and S2. Throughout the paper, in all the plots, we show only those data points that were actually included in the regression analysis; in other words the data for complexes for which the dominant interaction was not the halogen bond can be found only in the Supplementary Materials.

### 3.2. CCDC Data Search

The search of the relevant R–X⋅⋅⋅O=P interactions was performed using the CCDC 2020 offline data (program ConQuest 2.0.4). Search criteria: NM~X∙∙∙O~P(~NM)_3_ fragment, where (i) symbol ~ stands for any bond; (ii) X = Cl, Br, I; (iii) NM is any nonmetal; (iv) d(X∙∙∙O) ≡ *r* is less than the Bondi’s vdW radii sums; (v) ∠(NM~X∙∙∙O) ≡ *β*, 150° ≤ *β* ≤ 180°; (vi) number of bonded atoms for X is 1; (vii) number of bonded atoms for P is 4; (viii) structures are non-disordered; (ix) final R1 index [I ≥ 2σ (I)] is less or equal 10%.

## 4. Conclusions

In this work, we have considered a large set of 128 halogen-bonded complexes formed by trimethylphosphine oxide and halogen donors belonging to various classes of chemical compounds. The energies of these complexes span from 3.6 to 124 kJ/mol, while the halogen bond distances *R*, measured as a percentage of the sum of van der Waals radii of participating atoms, ranged from 100% down to 62%. The obtained distributions of interatomic distances and angles are rather similar to those obtained from the comprehensive search in the CCDC 2020 database of various RX⋅⋅⋅PO short contacts (compare Figure 1 and Figure 2). On the one hand, the Me_3_P=O molecule could be considered as a probe used to characterize the halogen-donating ability of isolated F-, Cl-, Br-, I- and At-containing species (the size of the σ-hole on halogen atom). On the other hand, the spectroscopic parameters of phosphine oxide involved in a R–X⋅⋅⋅O=P complex were used to determine the energy and geometry of the halogen bond. We showed that the change of ^31^P NMR chemical shift and P=O stretching frequency upon complexation have practically the same diagnostic value: they are well correlated with each other (Figure 10), linearly correlated to the halogen bond energy Δ*E* (Figure 7 and Figure 8) and exponentially related to halogen bond geometry (Figure 10). The overall spans of spectroscopic parameters are substantial: ca. 45 ppm for the ^31^P NMR chemical shift and ca. 50 cm^−1^ for the P=O frequency. Interestingly, the spectroscopic correlation with *R* values is general, i.e., it is fulfilled for the whole set of complexes at once, while in many other cases correlations remain halogen-specific, i.e., different for F-, Cl, Br-, I- or At-containing halogen donors. We believe that the interdependences between halogen bond descriptors and spectroscopic markers—Equations (5)–(9)—would be useful in case direct crystallographic or calorimetric data are not available, as in the case of halogen-bonded complexes in liquids, in solutions and in other kinds of disordered media.

## Figures and Tables

**Table 1:** The numerical values of fitted parameters *A*, *b*, *B*, *D*, *K* and *M* used in Equations (1)–(6). The resulting curves are shown in Figure 4, Figure 5, Figure 6 and Figure 7.

Donor Atom	Equations (1) and (3)	Equation (1)	Equation (3)	Equation (4)	Equation (5)	Equation (7)
	*A*, kJ/mol	*b*, Å^−1^	*B*	*D*	*K*, kJ/mol/cm^−1^	*M*, kJ/mol/ppm
F	5490	2.4	7.2	0.20	1.30	2.9
Cl	1470	1.5	4.9	0.25	0.75	2.45
Br	4100	1.8	6.1	0.30	0.85	2.65
I	16,300	2.2	7.7	0.34	0.90	3.05
At	138,000	2.95	10.6	0.35	1.00	3.30

**Table 2:** The numerical values of fitted parameters *C*_G_, *C*_V_, *C*_rho_ and *C*_Lap_ used in Equation (9) to fit data points shown in Figures S3–S6. For comparison, several correlation coefficients previously reported in [77] are added to the last two columns.

	This Work				Ref. [77]	
Halogen Donor	*C*_G_, A^3^	*C*_V_, A^3^	*C*_rho_, A^3^⋅kJ/mol/a.u.	*C*_Lap_, A^5^⋅kJ/mol/a.u.	*C*_G_, A^3^	*C*_V_, A^3^
F	0.18	0.14	600	150		
Cl	0.47	0.43	1550	320	0.47	0.49
Br	0.57	0.60	1300	400	0.57	0.58
I	0.74	0.70	1600	510	0.67	0.68
At	0.78	0.75	1900	530		

## Supplementary Materials

The following are available online at https://www.mdpi.com/1420-3049/25/6/1406/s1, Figure S1: optimized structures of Me_3_P=O⋅⋅⋅XR complexes, Table S1: calculated halogen bond geometries, complexation energies and spectroscopic parameters, Table S2: QTAIM parameters (*ρ*, ∇^2^*ρ*, *V* and *G*) at halogen bond BCP, Figure S2: the correlation between angles *β* (angle O⋅⋅⋅X–R) and the complexation energy, Table S3: geometric parameters of the R–X∙∙∙O=P halogen bonds found in CCDC 2020 database for X = Cl, Br, I, Figure S3: correlation between Δ*E* and local kinetic energy density *G* at BCP, Figure S4: correlation between Δ*E* and local potential energy density *V* at BCP, Figure S5: correlation between Δ*E* and electron density *ρ* at BCP, Figure S6: correlation between energy Δ*E* and Laplacian of electron density ∇^2^*ρ* at BCP, Figure S7: correlation between the extremal value of electrostatic potential *ESP*_max_ and change of the ^31^P NMR chemical shift upon complexation, ΔδP, for Me_3_P=O⋅⋅⋅XR complexes.
