# Supplementary material for: Phosphine Oxides as Spectroscopic Halogen Bond Descriptors: IR and NMR Correlations with Interatomic Distances and Complexation Energy
Source: Molecules. 2020 Mar 19;25(6):1406. doi: 10.3390/molecules25061406 (PMC7144381; doi:10.3390/molecules25061406)
Supplement: Supplementary file 1 [file molecules-25-01406-s001.pdf]

Supporting information for

Phosphine oxides as spectroscopic halogen bond descriptors: IR and NMR correlations with interatomic distances and complexation energy

A.S. Ostras', D.M. Ivanov, A.S. Novikov, P.M. Tolstoy

Institute of Chemistry, St. Petersburg State University, Russia

* – corresponding author, peter.tolstoy@spbu.ru

Contents **Page**

Figure S1. Optimized structures of complexes formed by $Me_3P{=}O$ with halogen-containing molecules. 2

Table S1. Calculated halogen bond geometries, complexation energies and spectroscopic parameters for complexes formed by $Me_3P{=}O$ with halogen-containing molecules. 10

Table S2. QTAIM parameters (ρ, $\nabla^2\rho$, V and G) at halogen bond critical point for complexes formed by $Me_3P{=}O$ with halogen-containing molecules. 13

Figure S2. The correlation between angles β (angle O···X–R) and the complexation energy for complexes formed by $Me_3P{=}O$ with halogen-containing molecules. 16

Table S3. Geometric parameters of the R–X···O=P halogen bonds found in CCDC 2020 database for X = Cl, Br. 17

Figure S3. Correlation between ΔE and local kinetic energy density G at BCP (3;–1) for $Me_3P{=}O{\cdots}X{-}R$ complexes. 19

Figure S4. Correlation between ΔE and local potential energy density V at BCP (3;–1) for $Me_3P{=}O{\cdots}X{-}R$ complexes. 19

Figure S5. Correlation between ΔE and electron density ρ at BCP (3;–1) for $Me_3P{=}O{\cdots}X{-}R$ complexes. 20

Figure S6. Correlation between energy ΔE and Laplacian of electron density $\nabla^2\rho$ at BCP (3;–1) for $Me_3P{=}O{\cdots}X{-}R$ complexes 20

Figure S1. Calculated (M06-2x/ def2-TZVPPD) optimized structures of 128 intermolecular 1:1 complexes formed by $Me_3P{=}O$ with halogen-containing molecules. The complexes in which the dominant intermolecular interaction is other than the halogen bond are marked in red color.

**halogens**				
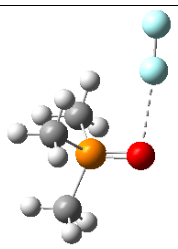	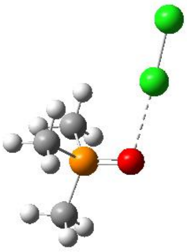	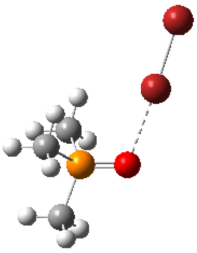	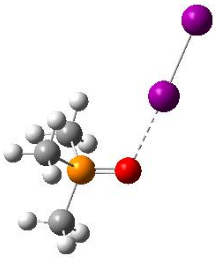	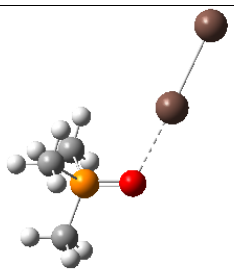
**[1] F_2**	**[2] Cl_2**	**[3] Br_2**	**[4] I_2**	**[5] At_2**

**interhalides**			
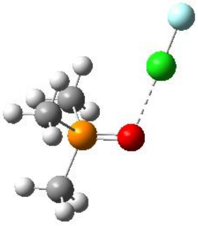	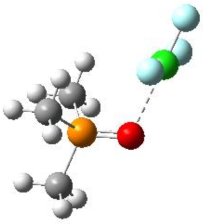	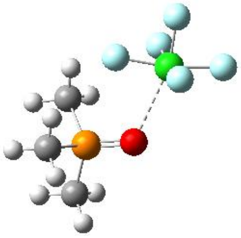	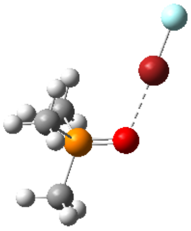
**[6] ClF**	**[7] ClF_3**	**[8] ClF_5**	**[9] BrF**
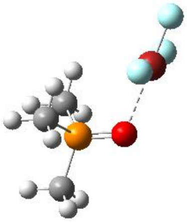	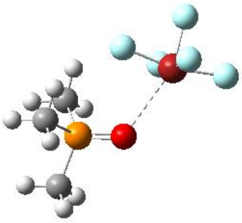	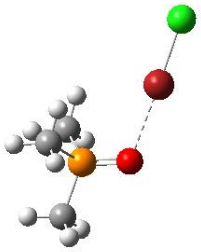	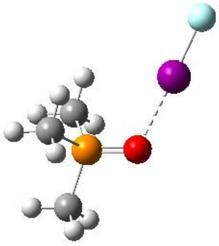
**[10] BrF_3**	**[11] BrF_5**	**[12] BrCl**	**[13] IF**
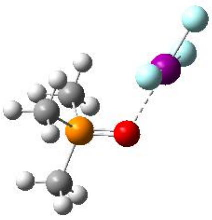	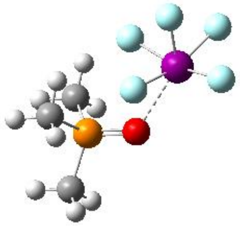	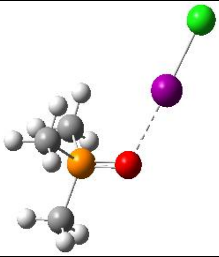	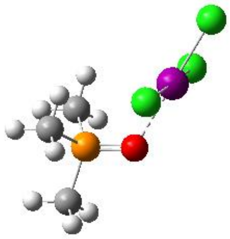
**[14] IF_3**	**[15] IF_5**	**[16] ICl**	**[17] ICl_3**
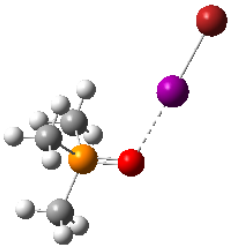	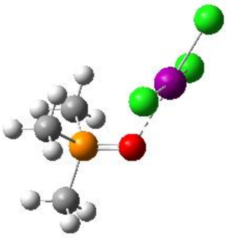	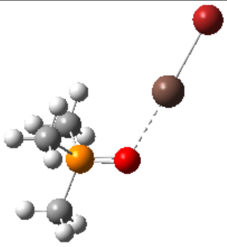	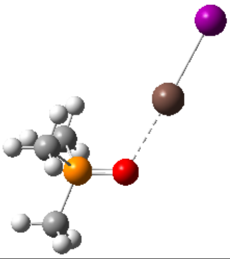
**[18] IBr**	**[19] AtCl**	**[20] AtBr**	**[21] AtI**

oxohalides			
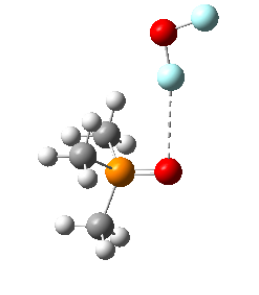	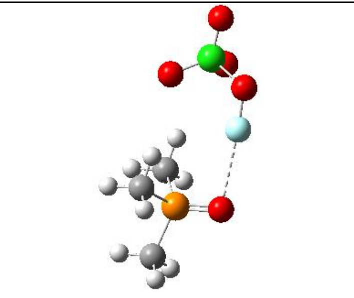	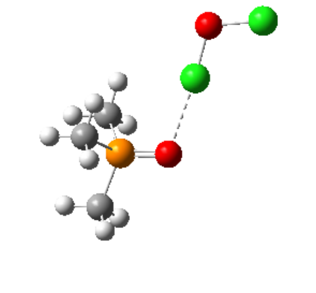	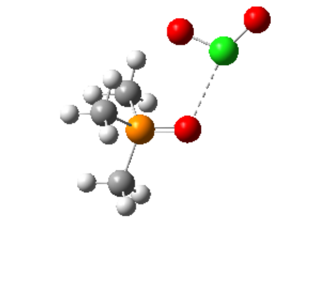
[22] OF_2	[23] ClO_3OF	[24] Cl_2O	[25] ClO_2
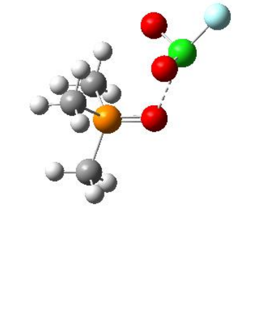	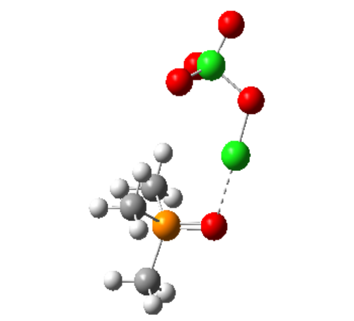	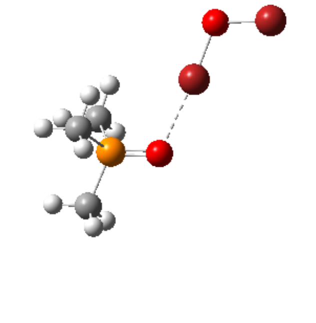	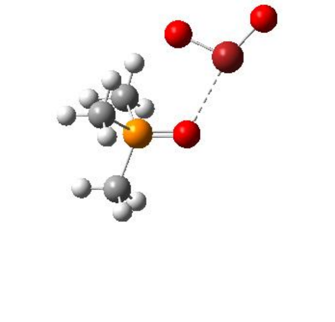
[26] ClO_2F	[27] ClO_3OCl	[28] Br_2O	[29] BrO_2
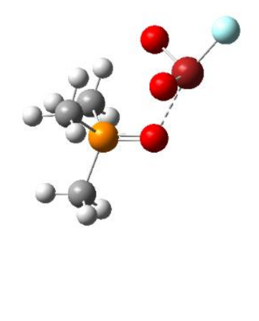	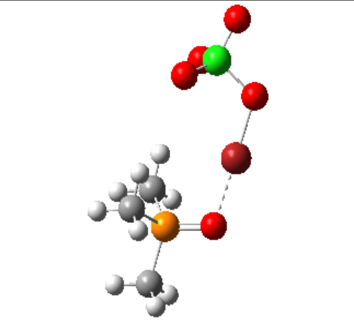	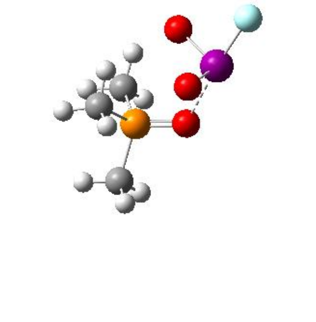	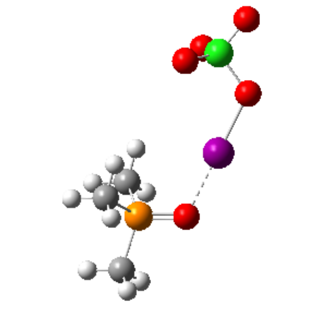
[30] BrO_2F	[31] ClO_3OBr	[32] IO_2F	[33] ClO_3OI

pseudohalides			
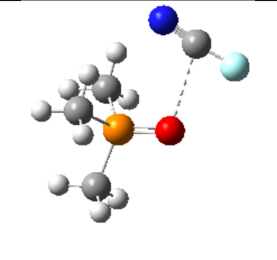	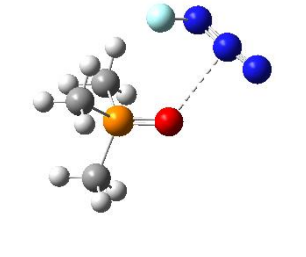	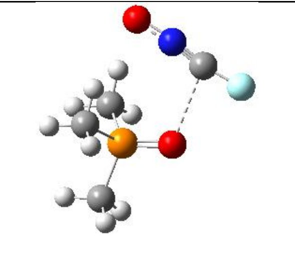	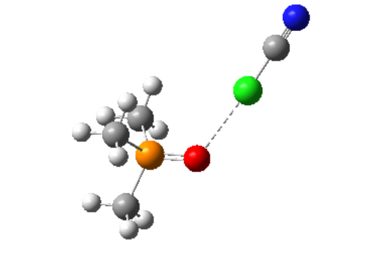
[34] FCN	[35] FN_3	[36] FCNO	[37] ClCN
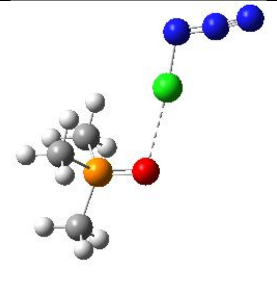	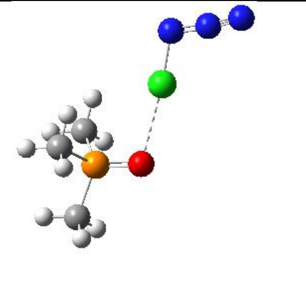	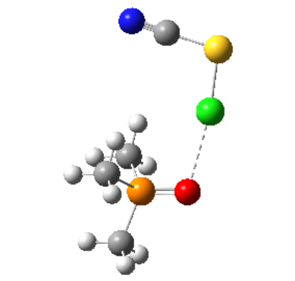	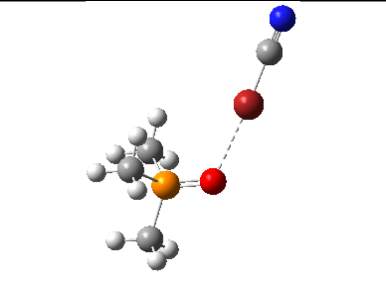
[38] ClN_3	[39] ClNCO	[40] ClSCN	[41] BrCN

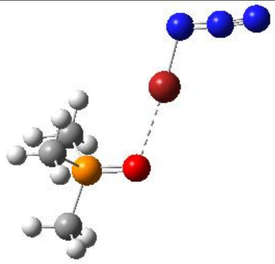	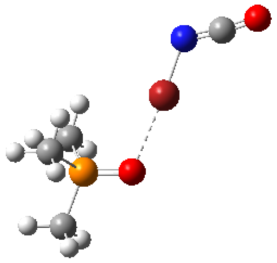	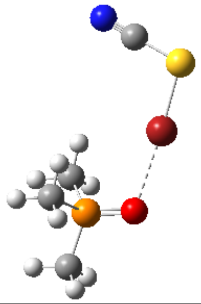	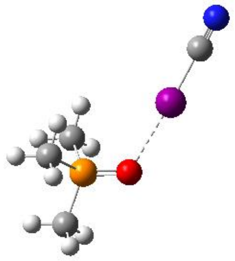
**[42] BrN_3**	**[43] BrNCO**	**[44] BrSCN**	**[45] ICN**

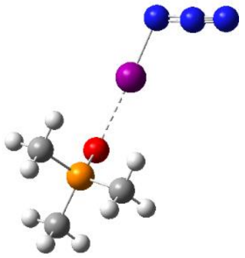	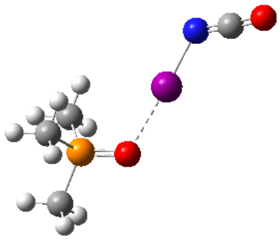	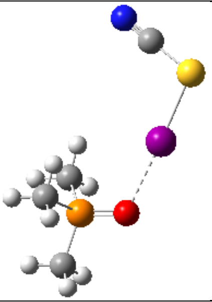
**[46] IN_3**	**[47] INCO**	**[48] ISCN**

**halogenated methanes and their derivatives**			
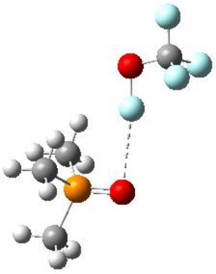	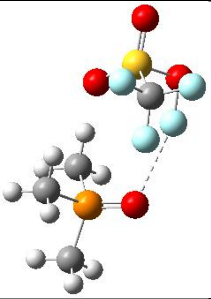	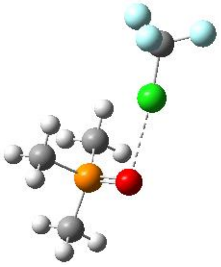	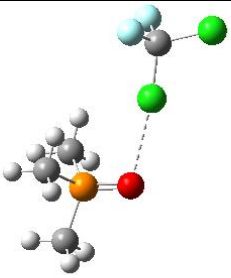
**[49] CF_3OF**	**[50] CF_3SO_2OF**	**[51] CF_3Cl**	**[52] CCl_2F_2**
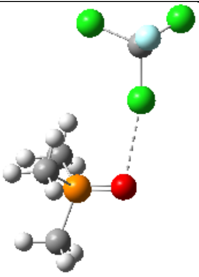	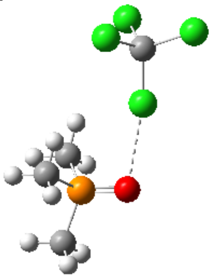	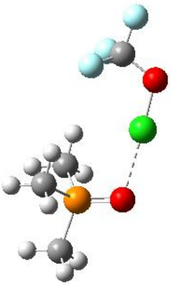	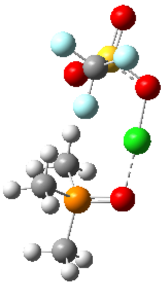
**[53] CCl_3F**	**[54] CCl_4**	**[55] CF_3OCl**	**[56] CF_3SO_2OCl**
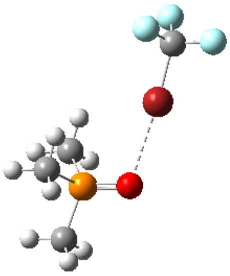	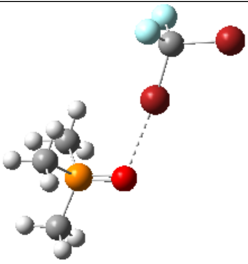	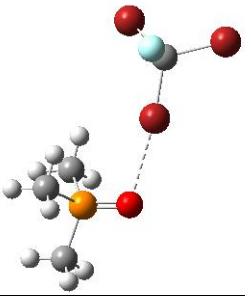	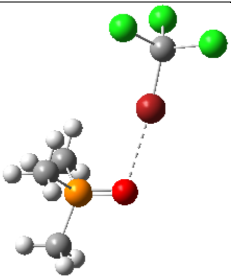
**[57] CF_3Br**	**[58] CBr_2F_2**	**[59] CBr_3F**	**[60] $CBrCl_3$**

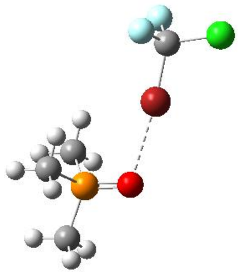	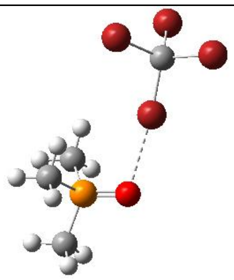	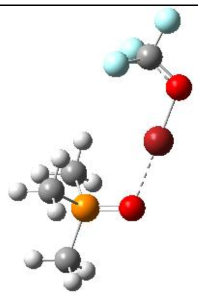	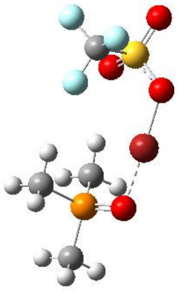
[61] $CBrClF_2$	[62] CBr_4	[63] CF_3OBr	[64] CF_3SO_2OBr
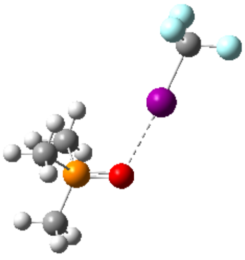	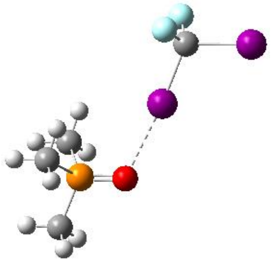	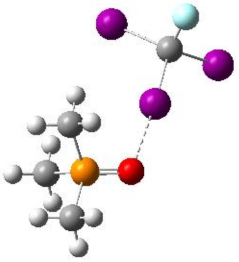	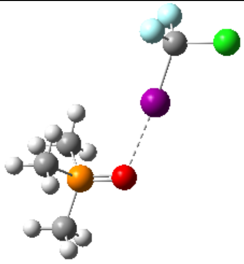
[65] CF_3I	[66] CI_2F_2	[67] CI_3F	[68] $CIClF_2$
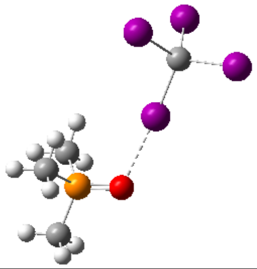			
[69] CI_4			

halogenated ethylene, halogenated acetylene and their derivatives			
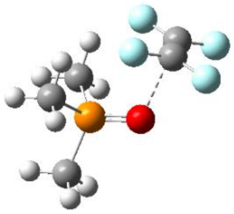	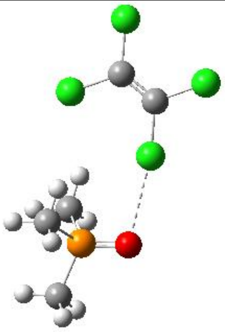	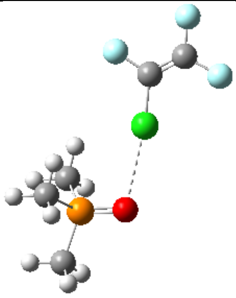	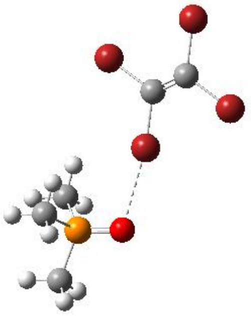
[70] C_2F_4	[71] C_2Cl_4	[72] C_2F_3Cl	[73] C_2Br_4
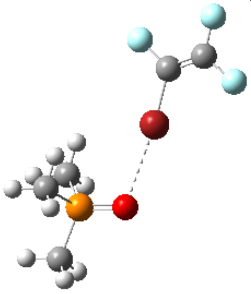	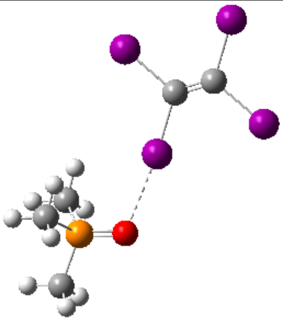	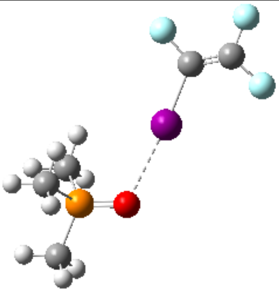	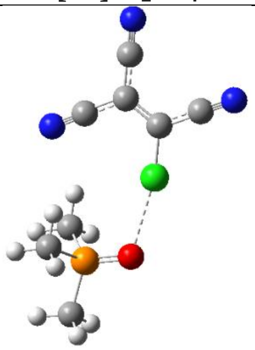
[74] C_2F_3Br	[75] C_2I_4	[76] C_2F_3I	[77] $C_2(CN)_3Cl$

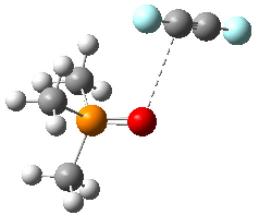	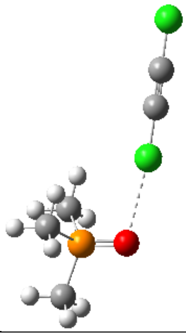
[78] C_2F_2	[79] C_2Cl_2

phosgene and its derivatives			
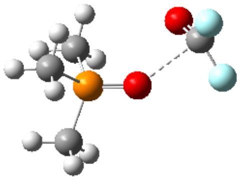	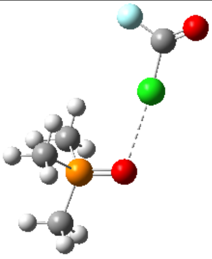	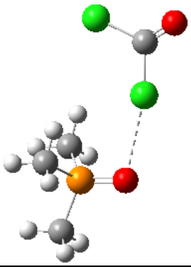	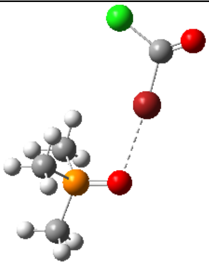
[80] COF_2	[81] COClF	[82] $COCl_2$	[83] COBrCl
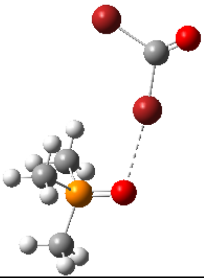	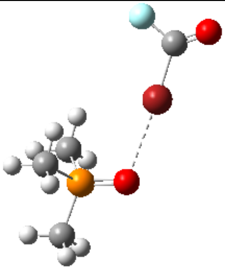	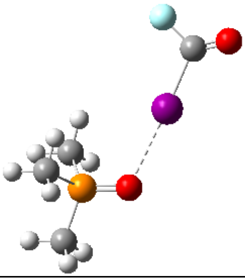	
[84] $COBr_2$	[85] COBrF	[86] COIF	

thionyl- and sulphurylhalides		
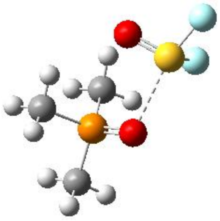	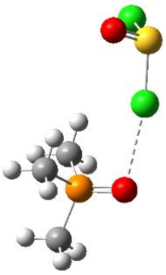	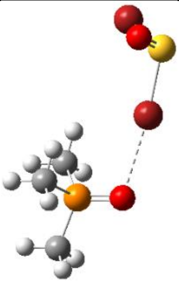
[87] SOF_2	[88] SO_2ClF	[89] $SOCl_2$
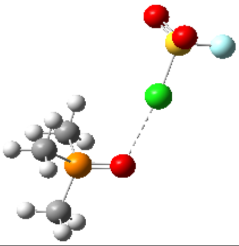	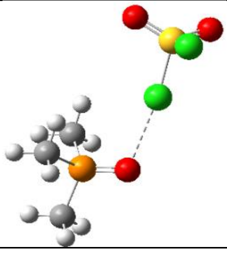	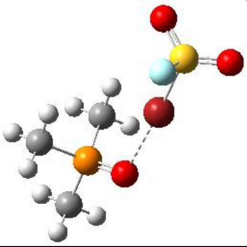
[90] $SOBr_2$	[91] SO_2Cl_2	[92] SO_2BrF

sulphur halides and sulphur hypohalites		
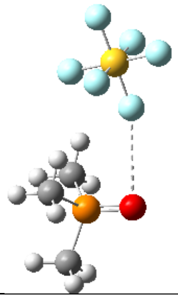	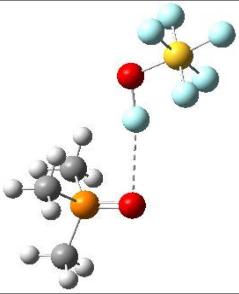	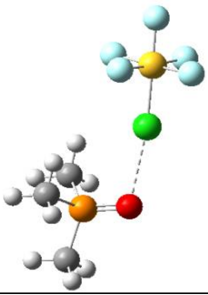
[93] SF_6	[94] SF_5OF	[95] SF_5Cl
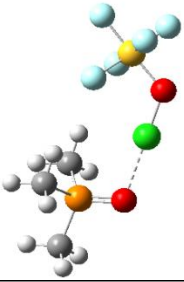	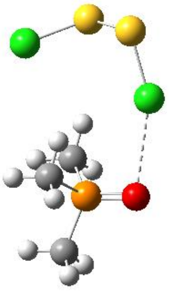	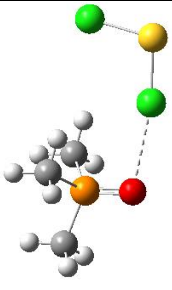
[96] SF_5OCl	[97] S_2Cl_2	[98] SCl_2
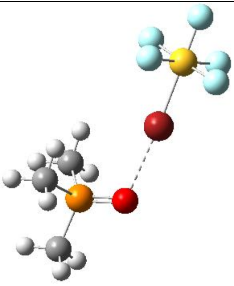	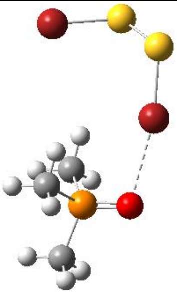	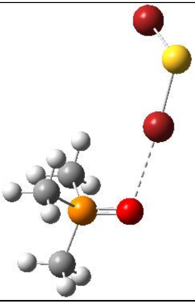
[99] SF_5Br	[100] S_2Br_2	[101] SBr_2

halogenated nitrogen-containing compounds			
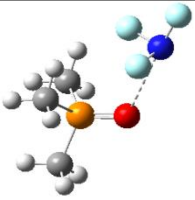	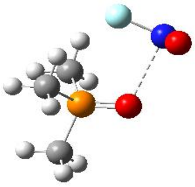	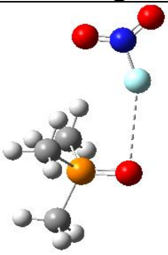	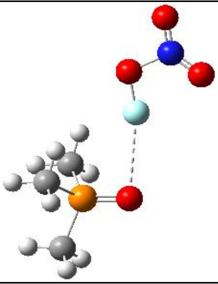
[102] NF_3	[103] NOF	[104] NO_2F	[105] NO_2OF
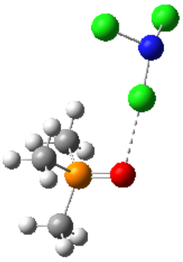	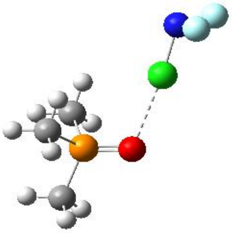	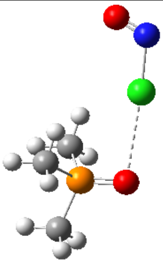	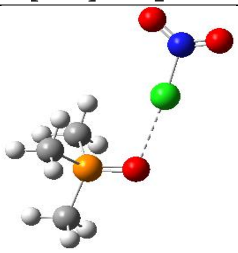
[106] NCl_3	[107] NF_2Cl	[108] NOCl	[109] NO_2Cl

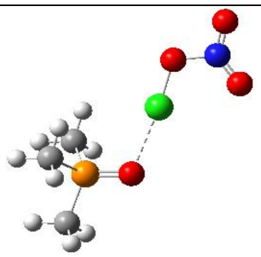	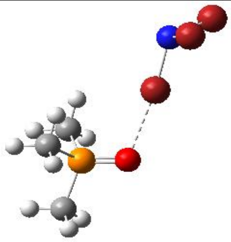	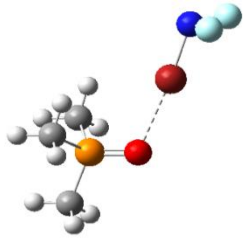	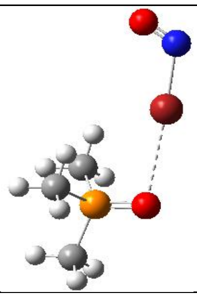
**[110] NO_2OCl**	**[111] NBr_3**	**[112] NF_2Br**	**[113] NOBr**

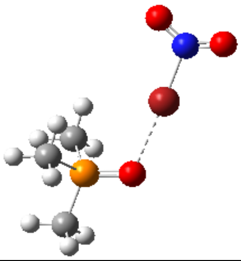	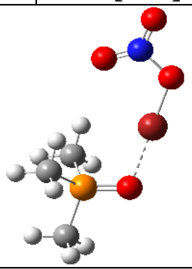	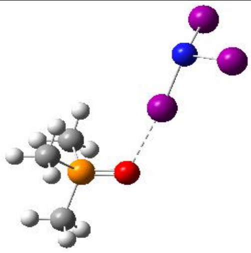
**[114] NO_2Br**	**[115] NO_2OBr**	**[116] NI_3**

**organic compounds**		
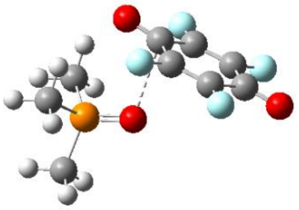	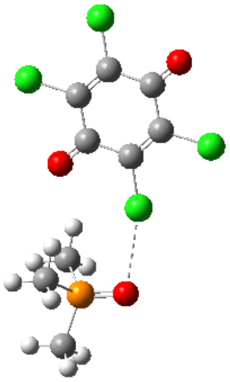	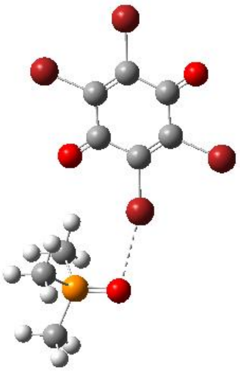
**[117] tetrafluoro-1,4-benzoquinone**	**[118] tetrachloro-1,4-benzoquinone**	**[119] tetrabromo-1,4-benzoquinone**
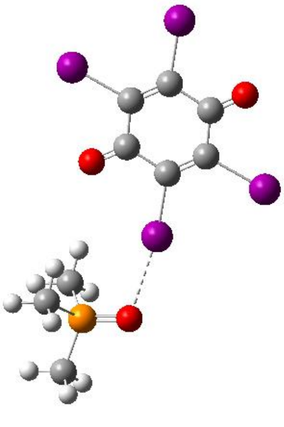	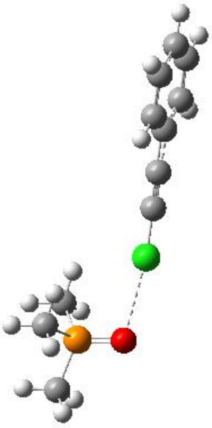	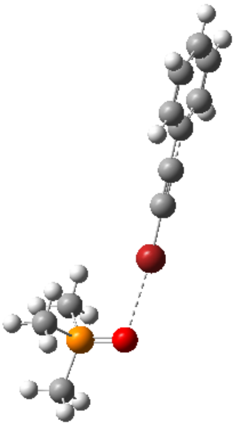
**[120] tetraiodo-1,4-benzoquinone**	**[121] $C_6H_5(C\equiv C)Cl$**	**[122] $C_6H_5(C\equiv C)Br$**

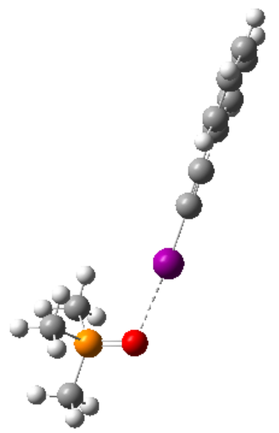	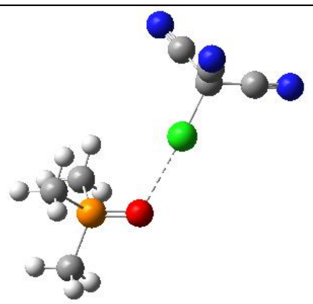	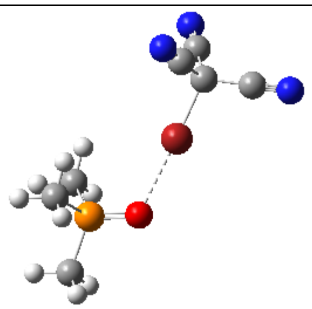
**[123] $C_6H_5(C{\equiv}C)I$**	**[124] $CCl(CN)_3$**	**[125] $CBr(CN)_3$**
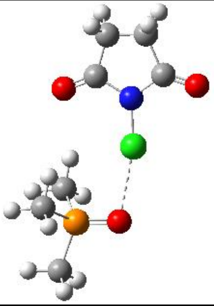	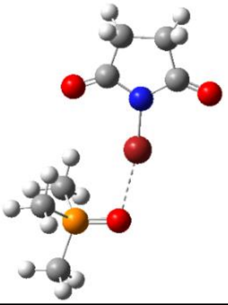	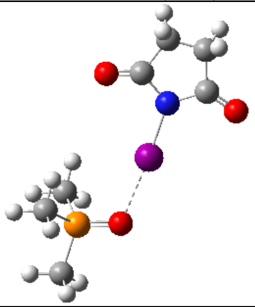
**[126] N-chlorosuccinimide**	**[127] N-bromosuccinimide**	**[128] N-iodosuccinimide**

Table S1. Results of quantum-chemical calculation (M06-2x/def2-TZVPPD) of optimized geometries, complexation energies (BSSE-corrected) and spectroscopic parameters of 128 intermolecular 1:1 complexes formed by $Me_3P{=}O$ with halogen-containing molecules. The complexes in which the dominant intermolecular interaction is other than the halogen bond are marked in red color. In ambiguous cases the halogen atoms which participate in halogen bond formation are highlighted in bold font. For the definitions of the parameters listed please refer to Scheme 1 in the main text.

№	Halogen donor	ESP_{max}, kJ/mol	ΔδP, ppm	Δν, cm^{-1}	ΔE, kJ/mol	*α* (P=O···X), °	*β* (O···X–R), °	*r*, Å
	**halogens**							
**1**	F_2	57.49	3.51	8.15	8.60	99.4	174.6	2.56
**2**	Cl_2	106.58	9.22	29.58	25.76	109.2	177.0	2.61
**3**	Br_2	79.54	12.13	42.15	33.64	112.7	177.8	2.62
**4**	I_2	112.61	17.09	53.4	42.34	117.2	177.9	2.67
**5**	At_2	152.51	16.33	56.5	51.3	119.2	177.9	2.67
	**interhalides**							
**6**	ClF	170.89	15.5	49.45	43.45	112.4	179.3	2.38
**7**	ClF_3	169.84	17.01	54.59	45.12	113.7	180.0	2.43
**8**	ClF_5	133.88	11.38	29.3	36.86	122.2	163.4	2.69
**9**	BrF	205.54	21.67	71.34	59.97	115.0	179.3	2.36
**10**	BrF_3	207.38	24.59	79.07	60.89	115.7	179.8	2.40
**11**	BrF_5	179.03	14.53	41.36	53.04	124.8	155.6	2.68
**12**	BrCl	140.96	13.55	46.81	38.61	113.7	178.3	2.57
**13**	IF	250.69	25.63	77.7	79.57	119.4	178.6	2.43
**14**	IF_3	239.14	30.54	95.66	77.37	120.2	179.8	2.43
**15**	IF_5	212.36	17.91	59.42	71.43	127.1	145.4	2.68
**16**	ICl	139.91	20.08	69.01	59.66	119.3	178.6	2.53
**17**	ICl_3	173.25	36.02	119.17	88.31	122.0	179.3	2.53
**18**	IBr	166.16	18.08	65.22	53.57	118.6	178.5	2.58
**19**	AtCl	239.25	22.99	68.99	76.14	121.4	178.5	2.54
**20**	AtBr	215.0	21.23	69.25	69.64	120.8	178.3	2.57
**21**	AtI	128.63	17.88	61.41	59.33	119.3	178.0	2.63
	**oxohalides**							
**22**	OF_2	28.35	1.15	1.61	3.58	95.4	169.2	2.91
**23**	ClO_3OF	45.15	3.76	8.09	10.04	101.9	176.1	2.67
**24**	Cl_2O	132.04	11.73	35.48	31.98	111.3	175.4	2.52
**25**	ClO_2	139.13	9.82	26.1	36.65	117.9	159.2	2.74
**26**	ClO_2F	179.81	12.14	35.41	46.33	117.1	158.5	2.58
**27**	ClO_3OCl	173.0	24.99	80.16	50.48	109.5	178.4	2.26
**28**	Br_2O	153.83	16.41	50.99	44.24	114.4	175.4	2.51
**29**	BrO_2	174.04	14.31	42.92	54.34	119.7	165.7	2.68
**30**	BrO_2F	217.35	22.96	86.47	75.40	118.5	165.0	2.40
**31**	ClO_3OBr	211.05	39.60	121.01	76.66	112.1	178.3	2.16
**32**	IO_2F	254.1	36.39	139.07	124.07	121.1	173.8	2.27
**33**	ClO_3OI	263.29	39.96	118.46	105.28	118.56	179.6	2.28
	**pseudohalides**							
**34**	FCN	116.55	9.19	23.39	36.80	---	---	2.65
**35**	FN_3	122.85	6.79	14.03	26.87	---	---	2.70
**36**	FCNO	115.24	11.29	34.68	40.74	---	---	2.57
**37**	ClCN	122.06	7.63	18.22	26.35	124.7	177.0	2.70
**38**	ClN_3	97.65	7.53	25.61	23.46	108.7	174.8	2.67
**39**	ClNCO	121.0	9.18	27.09	27.41	110.3	176.0	2.61
**40**	ClSCN	100.0	8.79	26.96	24.44	107.8	172.0	2.71

**41**	BrCN	175.88	8.84	27.08	33.94	121.2	176.9	2.71
**42**	BrN_3	126.78	11.92	38.85	34.34	113.4	175.7	2.63
**43**	BrNCO	153.04	13.44	43.86	40.48	114.7	176.6	2.56
**44**	BrSCN	129.15	12.66	41.38	34.65	113.0	176.4	2.66
**45**	ICN	207.38	13.61	42.04	50.70	123.8	177.7	2.68
**46**	IN_3	168.26	19.02	60.71	54.14	119.3	177.9	2.58
**47**	INCO	205.8	21.08	65.71	62.87	119.9	177.5	2.53
**48**	ISCN	170.63	20.02	66.95	54.38	118.8	177.8	2.59
**halogenated methanes and their derivatives**								
**49**	CF_3OF	28.09	2.00	4.75	5.25	95.2	172.1	2.77
**50**	CF_3SO_2OF	55.13	4.03	10.1	14.66	111.7	152.0	2.70
**51**	CF_3Cl	89.0	5.37	12.77	16.70	108.6	173.7	2.83
**52**	CCl_2F_2	85.84	5.61	14.90	16.89	108.1	173.0	2.82
**53**	CCl_3F	81.38	6.22	15.54	17.72	104.7	168.4	2.83
**54**	CCl_4	80.59	6.14	16.52	17.93	104.7	168.4	2.83
**55**	CF_3OCl	154.09	15.18	55.81	40.20	109.4	178.0	2.44
**56**	CF_3SO_2OCl	171.19	41.07	134.46	60.47	108.2	177.3	2.04
**57**	CF_3Br	103.43	6.75	20.68	22.74	112.2	175.9	2.83
**58**	CBr_2F_2	99.49	6.53	22.12	23.25	112.39	175.0	2.82
**59**	CBr_3F	97.13	8.56	23.93	24.13	109.7	172.6	2.81
**60**	$CBrCl_3$	96.86	8.20	24.88	24.32	110.3	174.2	2.79
**61**	$CBrClF_2$	99.75	6.91	21.52	23.04	111.6	174.2	2.82
**62**	CBr_4	96.34	8.41	25.99	24.97	109.7	174.0	2.78
**63**	CF_3OBr	191.89	23.76	88.61	86.78	113.6	179.4	2.35
**64**	CF_3SO_2OBr	220.0	44.03	141.39	87.13	111.3	177.1	2.11
**65**	CF_3I	131.78	10.07	32.29	34.79	117.9	176.6	2.81
**66**	CI_2F_2	121.28	11.06	35.38	34.75	117.2	175.4	2.80
**67**	CI_3F	119.18	13.25	40.29	35.73	116.4	176.1	2.77
**68**	$CIClF_2$	128.89	10.58	36.42	35.51	117.3	175.7	2.79
**69**	CI_4	118.91	13.50	42.88	37.37	116.7	176.3	2.74
**halogenated ethylene, halogenated acetylene and their derivatives**								
**70**	C_2F_4	86.89	4.52	11.28	19.42	---	---	2.78
**71**	C_2Cl_4	76.13	5.32	15.31	18.47	104.1	170.6	2.86
**72**	C_2F_3Cl	86.83	5.02	14.75	16.12	107.7	172.6	2.85
**73**	C_2Br_4	94.24	7.90	21.93	22.69	108.7	172.0	2.84
**74**	C_2F_3Br	105.53	7.07	21.93	22.68	111.8	174.4	2.84
**75**	C_2I_4	115.76	10.15	35.81	33.99	115.2	174.4	2.81
**76**	C_2F_3I	136.76	9.58	32.39	35.02	117.3	175.5	2.82
**77**	$C_2(CN)_3Cl$	148.05	8.72	24.76	26.23	119.6	168.8	2.89
**78**	C_2F_2	39.64	1.57	3.95	8.24	---	---	2.98
**79**	C_2Cl_2	96.1	5.26	13.32	17.41	108.5	173.9	2.82
**phosgene and its derivatives**								
**80**	COF_2	173.0	7.61	18.14	34.90	---	---	2.49
**81**	COClF	98.44	5.34	14.11	17.40	111.9	175.1	2.82
**82**	$COCl_2$	84.79	5.63	15.95	16.80	105.4	168.5	2.85
**83**	COBrCl	69.04	6.83	21.78	21.93	111.6	175.5	2.84
**84**	$COBr_2$	95.03	7.18	24.03	22.65	110.1	172.9	2.83
**85**	COBrF	119.7	6.63	20.31	23.41	114.1	176.6	2.83
**86**	COIF	137.29	9.90	33.3	36.08	119.3	177.7	2.81
**thionyl- and sulphurylhalides**								
**87**	SOF_2	181.92	12.27	39.3	43.35	---	---	2.61
**88**	SO_2ClF	123.64	8.00	23.71	24.45	116.5	173.7	2.71
**89**	$SOCl_2$	6.56	6.15	17.54	16.52	104.8	169.9	2.86
**90**	$SOBr_2$	9.19	7.90	26.43	21.99	109.0	174.2	2.85
**91**	SO_2Cl_2	109.46	8.92	21.01	23.50	112.9	172.1	2.72
**92**	SO_2BrF	139.91	10.95	35.44	36.82	117.9	175.6	2.70
**sulphur halides and sulphur hypohalites**								
**93**	SF_6	8.4	0.62	2.78	4.18	---	---	3.14
**94**	SF_5OF	29.14	1.91	4.42	5.46	95.3	170.6	2.76
**95**	SF_5Cl	94.24	8.20	22.41	21.16	107.2	172.7	2.73

**96**	SF_5OCl	158.81	18.13	55.55	43.54	119.3	179.8	2.37
**97**	S_2Cl_2	27.3	4.86	15.70	16.70	99.5	155.9	2.95
**98**	SCl_2	68.25	5.73	16.76	17.03	103.8	169.7	2.83
**99**	SF_5Br	115.24	11.35	36.17	34.15	114.02	176.8	2.68
**100**	S_2Br_2	96.6	8.23	27.72	22.85	106.8	168.5	2.83
**101**	SBr_2	87.94	9.02	27.69	24.34	110.3	176.4	2.78
**halogenated nitrogen-containing inorganic compounds**								
**102**	NF_3	71.66	2.47	4.56	10.92	---	---	2.89
**103**	NOF	104.48	9.39	26.59	35.23	---	---	2.58
**104**	NO_2F	80.33	0.80	2.42	3.95	---	---	3.06
**105**	NO_2OF	31.0	1.79	2.59	3.97	97.1	166.3	2.87
**106**	NCl_3	105.26	9.15	27.47	25.25	107.7	176.6	2.65
**107**	NF_2Cl	118.91	8.11	24.48	25.20	112.1	174.8	2.65
**108**	NOCl	25.73	2.07	9.03	6.62	99.7	176.1	3.07
**109**	NO_2Cl	106.84	9.75	31.75	26.93	111.7	176.1	2.61
**110**	NO_2OCl	154.1	13.05	39.06	35.97	114.1	174.8	2.47
**111**	NBr_3	120.75	11.30	39.72	33.94	113.5	173.8	2.63
**113**	NF_2Br	135.45	11.00	37.49	34.26	115.2	175.9	2.64
**114**	NOBr	17.06	2.80	11.36	9.11	103.1	176.9	3.1
**115**	NO_2Br	126.0	13.45	47.13	36.72	115.2	177.5	2.59
**116**	NO_2OBr	189.0	26.80	86.13	60.05	112.9	179.9	2.32
**117**	NI_3	148.58	18.28	57.86	49.21	118.50	174.6	2.61
**assorted organic compounds**								
**117**	tetrafluoro-1,4-benzoquinone	138.86	13.69	41.38	53.0	---	---	2.61
**118**	tetrachloro-1,4-benzoquinone	80.1	7.95	18.75	22.52	102.9	163.0	2.87
**119**	tetrabromo-1,4-benzoquinone	97.39	9.04	22.71	27.18	105.3	164.1	2.87
**120**	tetraiodo-1,4-benzoquinone	121.0	11.10	34.35	35.71	112.4	171.2	2.83
**121**	$C_6H_5C_2\mathbf{Cl}$	80.33	5.46	12.97	15.89	106.8	173.1	2.85
**122**	$C_6H_5C_2\mathbf{Br}$	102.64	7.27	18.70	22.65	111.0	175.1	2.84
**123**	$C_6H_5C_2\mathbf{I}$	134.14	10.68	30.80	34.88	116.7	176.5	2.82
**124**	$C\mathbf{Cl}(CN)_3$	147.53	9.58	28.24	29.54	119.0	176.7	2.63
**125**	$C\mathbf{Br}(CN)_3$	172.46	13.47	47.29	41.71	119.8	178.1	2.59
**126**	N-chlorosuccinimide	126	13.08	41.68	38.83	109.0	169.9	2.64
**127**	N-bromosuccinimide	96.34	9.73	27.5	28.54	105.0	166.9	2.67
**128**	N-iodosuccinimide	172.73	19.99	61.63	57.52	117.4	175.9	2.58

Table S2. The results of QTAIM analysis of electron density in intermolecular bond critical point (3; –1) for 1:1 complexes formed by $Me_3P{=}O$ with halogen-containing molecules. ρ, $\nabla^2\rho$, V and G are, respectively, electron density, Laplacian of electron density, local electron potential and kinetic energies densities at BCP. The complexes in which the dominant intermolecular interaction is other than the halogen bond are marked in red color. In ambiguous cases the halogen atoms which participate in halogen bond formation are highlighted in bold font.

№	Halogen bond donor	G, kJ/mol	V, kJ/mol	ρ, a.u.	$\nabla^2\rho$, a.u.
			**halogens**		
**1**	F_2	42.13	-33.02	0.0147	0.0781
**2**	Cl_2	56.43	-50.57	0.0231	0.0949
**3**	Br_2	61.61	-58.58	0.0270	0.0985
**4**	I_2	62.57	-63.28	0.0288	0.0942
**5**	At_2	70.69	-70.05	0.0297	0.1090
			**interhalides**		
**6**	**Cl**F	90.94	-91.733	0.0372	0.1373
**7**	**Cl**F$_3$	88.03	-88.58	0.0375	0.1333
**8**	**Cl**F$_5$	55.01	-50.67	0.0230	0.0904
**9**	**Br**F	101.48	-113.47	0.0455	0.1363
**10**	**Br**F$_3$	99.88	-110.36	0.0465	0.1362
**11**	**Br**F$_5$	60.60	-57.53	0.0267	0.0970
**12**	**Br**Cl	68.21	-66.47	0.0297	0.1066
**13**	**I**F	100.35	-116.74	0.0448	0.1279
**14**	**I**F$_3$	104.48	-127.49	0.0513	0.1241
**15**	**I**F$_5$	66.58	-67.49	0.0313	0.1001
**16**	**I**Cl	81.27	-88.70	0.0369	0.1125
**17**	**I**Cl$_3$	120.83	-156.92	0.0610	0.1291
**18**	**I**Br	74.74	-79.67	0.0342	0.1064
**19**	**At**Cl	94.32	-98.80	0.0378	0.1377
**20**	**At**Br	88.46	-91.64	0.0360	0.1306
**21**	**At**I	76.57	-77.11	0.0318	0.1163
			**oxohalides**		
**22**	O**F**$_2$	17.24	-11.73	0.0068	0.0346
**23**	ClO$_3$O**F**	81.62	-109.19	0.0417	0.0823
**24**	**Cl**$_2$O	68.31	-63.84	0.0275	0.1109
**25**	**Cl**O$_2$	45.26	-39.29	0.0184	0.0780
**26**	**Cl**O$_2$F	61.23	-56.85	0.0258	0.0999
**27**	ClO$_3$O**Cl**	117.66	-132.53	0.0530	0.0157
**28**	**Br**$_2$O	76.81	-77.49	0.0333	0.1160
**29**	**Br**O$_2$	55.88	-51.68	0.0241	0.0915
**30**	**Br**O$_2$F	93.95	-104.43	0.0461	0.1271
**31**	ClO$_3$O**Br**	151.99	-210.22	0.0761	0.1428
**32**	**I**O$_2$F	136.38	-188.17	0.0690	0.1289
**33**	ClO$_3$O**I**	143.28	-185.96	0.0642	0.1533
			**pseudohalides**		
**34**	**F**CN	34.37	-29.05	0.0155	0.0605
**35**	**F**N$_3$	34.21	-27.96	0.0132	0.0616
**36**	**F**CNO	42.31	-37.42	0.0192	0.0719
**37**	**Cl**CN	45.74	-38.70	0.0181	0.0804
**38**	**Cl**N$_3$	45.74	-38.70	0.0181	0.0804
**39**	**Cl**NCO	56.30	-50.15	0.0225	0.0951
**40**	**Cl**SCN	46.31	-40.02	0.0194	0.0801
**41**	**Br**CN	51.36	-46.00	0.0215	0.0864

**42**	BrN_3	60.07	-56.56	0.0258	0.0969
**43**	$BrNCO$	68.71	-66.65	0.0292	0.1078
**44**	$BrSCN$	57.20	-53.39	0.0251	0.0929
**45**	ICN	61.56	-60.47	0.0269	0.0954
**46**	IN_3	74.12	-78.44	0.0334	0.1064
**47**	$INCO$	82.03	-89.04	0.0365	0.1143
**48**	$ISCN$	72.58	-76.72	0.0335	0.1042
**halogenated methanes and their derivatives**					
**49**	CF_3OF	24.85	-17.84	0.0093	0.0485
**50**	CF_3SO_2OF	29.29	-21.46	0.0106	0.0566
**51**	CF_3Cl	35.33	-28.77	0.0149	0.0638
**52**	CCl_2F_2	35.92	-29.39	0.0152	0.0647
**53**	CCl_3F	35.73	-29.31	0.0153	0.0642
**54**	CCl_4	37.11	-30.69	0.0158	0.0663
**55**	CF_3OCl	81.20	-79.65	0.0333	0.1261
**56**	CF_3SO_2OCl	177.74	-243.73	0.0898	0.1702
**57**	CF_3Br	40.26	-34.85	0.0176	0.0696
**58**	CBr_2F_2	41.37	-36.03	0.0181	0.0712
**59**	CBr_3F	42.38	-37.17	0.0186	0.0725
**60**	$CBrCl_3$	43.57	-38.40	0.0191	0.0743
**61**	$CBrClF_2$	40.92	-35.58	0.0180	0.0705
**62**	CBr_4	44.42	-39.29	0.0194	0.0755
**63**	CF_3OBr	103.95	-118.31	0.0477	0.1364
**64**	CF_3SO_2OBr	220.31	-362.23	0.1116	0.1194
**65**	CF_3I	48.50	-45.43	0.0219	0.0786
**66**	CI_2F_2	49.67	-46.94	0.0225	0.0798
**67**	CI_3F	51.90	-11.87	0.0235	0.0825
**68**	$CIClF_2$	49.88	-47.13	0.0225	0.0801
**69**	CI_4	54.89	-53.28	0.0248	0.0861
**halogenated ethylene, halogenated acetylene and their derivatives**					
**70**	C_2F_4	27.55	-23.40	0.0130	0.0483
**71**	C_2Cl_4	33.70	-27.31	0.0144	0.0611
**72**	C_2F_3Cl	33.65	-27.11	0.0142	0.0612
**73**	C_2Br_4	39.72	-34.29	0.0174	0.0688
**74**	C_2F_3Br	39.62	-34.06	0.0172	0.0688
**75**	C_2I_4	48.60	-45.59	0.0219	0.0786
**76**	C_2F_3I	47.65	-44.27	0.0213	0.0777
**77**	$C_2(CN)_3Cl$	48.94	-42.33	0.0196	0.0846
**78**	C_2F_2	17.61	-14.06	0.0085	0.0322
**79**	C_2Cl_2	36.00	-29.34	0.0149	0.0650
**phosgene and its derivatives**					
**80**	COF_2	44.16	-38.36	0.0191	0.0761
**81**	$COClF$	36.21	-29.63	0.0152	0.0652
**82**	$COCl_2$	34.21	-27.90	0.0147	0.0617
**83**	$COBrCl$	39.86	-34.57	0.0176	0.0688
**84**	$COBr_2$	40.24	-35.04	0.0179	0.0693
**85**	$COBrF$	40.34	-34.93	0.0176	0.0697
**86**	$COIF$	48.29	-45.10	0.0217	0.0784
**thionyl- and sulphurylhalides**					
**87**	SOF_2	53.58	-51.00	0.0248	0.0856
**88**	SO_2ClF	45.70	-39.11	0.0189	0.0797
**89**	$SOCl_2$	33.35	-27.23	0.0148	0.0601
**90**	$SOBr_2$	39.34	-34.32	0.0180	0.0676
**91**	SO_2Cl_2	45.33	-38.91	0.0189	0.0788
**92**	SO_2BrF	53.11	-48.61	0.0231	0.0877
**sulphur halides and sulphur hypohalites**					
**93**	SF_6	9.89	-6.37	0.0044	0.0204
**94**	SF_5OF	25.68	-18.50	0.0096	0.0501
**95**	SF_5Cl	44.21	-37.79	0.0186	0.0771
**96**	SF_5OCl	93.64	-96.07	0.0393	0.1390

**97**	S_2**Cl_2**	27.92	-22.20	0.0128	0.0513
**98**	S**Cl_2**	35.62	-29.25	0.0154	0.0640
**99**	SF_5**Br**	55.19	-51.06	0.0241	0.0904
**100**	S_2**Br_2**	40.78	-35.74	0.0185	0.0698
**101**	S**Br_2**	44.26	-39.17	0.0196	0.0752
**halogenated nitrogen-containing inorganic compounds**					
**102**	N**F_3**	21.45	-16.87	0.0089	0.0397
**103**	NO**F**	41.62	-35.72	0.0175	0.0724
**104**	NO_2**F**	12.02	-7.96	0.0053	0.0245
**105**	NO_2O**F**	19.00	-13.03	0.0074	0.0380
**106**	N**Cl_3**	52.89	-46.91	0.0217	0.0897
**107**	NF_2**Cl**	51.92	-45.71	0.0211	0.0886
**108**	NO**Cl**	20.70	-15.73	0.0101	0.0391
**109**	NO_2**Cl**	56.38	-50.92	0.0233	0.0942
**110**	NO_2O**Cl**	75.82	-72.58	0.0306	0.1204
**111**	N**Br_3**	60.73	-57.38	0.0262	0.0976
**112**	NF_2**Br**	59.02	-55.42	0.0255	0.0954
**113**	NO**Br**	22.99	-18.58	0.0115	0.0417
**114**	NO_2**Br**	65.01	-62.86	0.0286	0.1023
**115**	NO_2O**Br**	109.93	-128.72	0.0515	0.1389
**116**	N**I_3**	70.60	-73.71	0.0318	0.1028
**assorted organic compounds**					
**117**	tetrafluoro-1,4-benzoquinone	41.03	-35.03	0.0177	0.0717
**118**	tetrachloro-1,4-benzoquinone	33.55	-27.27	0.0145	0.0607
**119**	tetrabromo-1,4-benzoquinone	38.20	-32.94	0.0171	0.0662
**120**	tetraiodo-1,4-benzoquinone	102.57	-76.79	0.0614	0.1155
**121**	$C_6H_5C_2$**Cl**	33.53	-26.99	0.0140	0.0610
**122**	$C_6H_5C_2$**Br**	39.51	-33.82	0.0170	0.0688
**123**	$C_6H_5C_2$**I**	47.84	-44.31	0.0211	0.0783
**124**	C**Cl**$(CN)_3$	54.42	-48.16	0.0220	0.0924
**125**	C**Br**$(CN)_3$	65.88	-63.24	0.0284	0.1044
**126**	N-chlorosuccinimide	59.88	-56.40	0.0257	0.0965
**127**	N-bromosuccinimide	50.44	-44.25	0.0206	0.0863
**128**	N-iodosuccinimide	75.57	-80.12	0.0337	0.1082

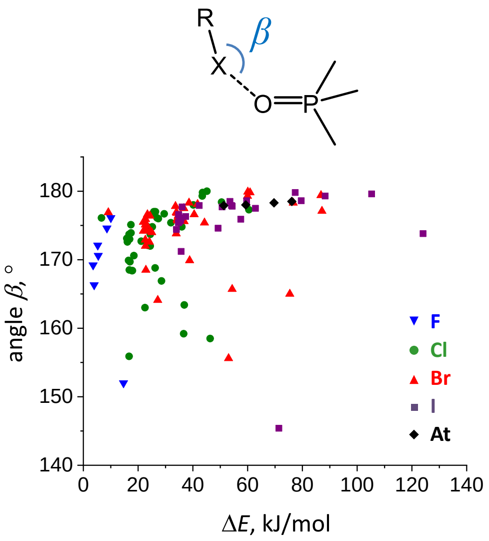

Figure S2. The correlation between angles β (angle O···X–R) and the complexation energy for 128 complexes studied in this work (X = F, Cl, Br, I, At). Stronger complexes seem to be characterized by generally smaller range of β angles closer to 180°.

Table S3. Geometric parameters of the R–X···O=P halogen bonds found in CCDC 2020 database for X = Cl, Br. r – X...O distance; R – normalized distance parameter $R = r/(R_O + R_X)$, where R_O and R_X are van-der-Waals radii of oxygen and halogen atoms, respectively, α and β – X···O=P and R–X···O angles, respectively. Search criteria: NM~X···O~P(~NM)$_3$ fragment, where (i) ~ is any bond; (ii) X = Cl, Br, I; (iii) NM is any nonmetal; (iv) d(X···O) ≡ r is less than the Bondi's vdW sums; (v) ∠(NM~X···O) ≡ β, 150° ≤ β ≤ 180°; (vi) number of bonded atoms for X is 1; (vii) number of bonded atoms for P is 4; (viii) structures are non-disordered; (ix) Final R_1 index [I≥2σ (I)] is less or equal 10%.

CCDC code	r, Å	R	α, °	β, °	CCDC code	r, Å	R	α, °	β, °
X = Cl									
AYEQOK	3.084	0.94	149.2	165.8	MAQPOJ	3.215	0.98	110.1	162.7
CAZLAT	3.037	0.93	138.1	159.9	MYPOCL	3.198	0.98	147.6	159.8
CEPSIA	2.896	0.89	120.2	170.4	NEKMUM	3.190	0.98	101.8	173.9
	2.923	0.89	128.4	165.1	NIMBUG	3.260	1.00	115.6	161.3
DAVKUG	3.180	0.97	131.8	161.5	NIYYIG	3.046	0.93	132.3	169.2
DUYXUQ	3.203	0.98	121.1	168.8	OBUCAQ	3.236	0.99	173.2	171.9
EHENIO	2.817	0.86	107.9	180.0	OCETEX	3.044	0.93	132.2	167.3
FIJROH	3.194	0.98	153.1	161.6	ROBROP	3.269	1.00	119.4	170.0
HOHNOH	2.929	0.90	144.9	171.3	RONOXN	3.245	0.99	125.7	171.0
HOHREB	3.062	0.94	140.7	173.2	RUNZAD	3.018	0.92	137.7	151.1
IKENIV	3.140	0.96	117.8	164.0	SERXAO	3.240	0.99	125.1	165.1
JANNAO	3.218	0.98	104.3	168.6	TUQZUA	2.890	0.88	120.6	168.2
KANSUO	3.183	0.97	156.8	153.5	WIXVII	2.988	0.91	120.8	171.2
KOSYIA	3.237	0.99	109.9	176.2	XUCSEU	3.232	0.99	129.8	158.1
LOGZUF	3.257	1.00	116.6	170.0	YIVPAV	2.996	0.92	132.2	175.1
MANNOH	3.091	0.95	127.0	172.1	VEPJEI	3.120	0.95	111.4	171.4
X = Br									
NEBXOK	3.035	0.90	115.2	176.3	PUVGUJ	2.975	0.88	110.9	166.7
BCMOPH	3.106	0.92	155.6	165.5		2.805	0.83	116.9	171.6
BILBUV	3.196	0.95	99.7	173.6	SIJSOU	3.034	0.90	102.1	171.5
CAKXIW	3.294	0.98	111.7	159.6	TANXAH	3.255	0.97	114.8	177.2
COJXAB	2.948	0.87	121.9	163.2	TOTNOH	3.239	0.96	126.5	162.0
CORFIZ	2.951	0.88	113.2	168.5		3.274	0.97	126.3	162.0
FEFBOI	2.875	0.85	128.8	174.8	UGISEI	3.348	0.99	104.3	157.2
FUGCAN	3.027	0.90	146.7	169.2	UXOBUG	2.802	0.83	151.9	170.6
ISIHAT	3.021	0.90	163.8	166.1		2.788	0.83	149.5	168.7
JOTVIX	2.970	0.88	160.0	166.7	VIZGUG	3.200	0.95	149.8	161.2
MUMKOV	3.046	0.90	117.8	176.8	VUJKOA	3.042	0.90	124.4	175.0
NUCYAL	3.093	0.92	105.3	167.9	XEGSUZ	3.215	0.95	131.7	169.6
OBULAZ	2.983	0.89	134.0	176.6	YEFWIQ	2.967	0.88	126.8	165.6
PASDIX	2.742	0.81	153.6	166.1	ZUFPEX	2.798	0.83	145.0	175.3
PASLAY	2.862	0.85	129.6	173.2		2.811	0.83	117.2	175.1
	2.893	0.86	127.8	169.7	VEPJEI	3.345	0.99	109.4	168.2

Table S3 continued. Geometric parameters of the R–X···O=P halogen bonds found in CCDC 2020 database for X = I. *r* – X...O distance; *R* – normalized distance parameter $R = r/(R_O + R_X)$, where R_O and R_X are van-der-Waals radii of oxygen and halogen atoms, respectively, α and β – X···O=P and R–X···O angles, respectively. Search criteria: NM~X···O~P(~NM)$_3$ fragment, where (i) ~ is any bond; (ii) X = Cl, Br, I; (iii) NM is any nonmetal; (iv) d(X···O) ≡ r is less than the Bondi's vdW sums; (v) ∠(NM~X···O) ≡ β, 150° ≤ β ≤ 180°; (vi) number of bonded atoms for X is 1; (vii) number of bonded atoms for P is 4; (viii) structures are non-disordered; (ix) Final R_1 index [I≥2σ (I)] is less or equal 10%.

CCDC code	*r*, Å	*R*	α, °	*β*, °	CCDC code	*r*, Å	*R*	α, °	*β*, °
X = I									
ANUQAC	2.752	0.79	158.6	174.6	MELCEM	2.894	0.83	161.0	178.1
COLCOY	2.904	0.83	133.3	166.9	QECCUW	3.018	0.86	124.6	174.9
COLCUE	2.965	0.85	135.5	169.6	ROHCEW	3.009	0.86	129.3	176.6
ECUQUO	2.835	0.81	139.0	170.6	SAXJUZ	2.683	0.77	172.2	176.8
	2.864	0.82	125.0	177.0	SIJSUA	3.098	0.89	106.7	169.9
GIDRES	2.809	0.80	114.1	173.1	SOKKUB	2.936	0.84	163.0	165.4
GULYOB	2.760	0.79	136.3	174.0		3.204	0.92	112.6	161.3
	2.862	0.82	126.8	168.1	SOKLAI	2.992	0.85	126.3	165.3
HARREA	3.217	0.92	116.4	169.2	SOKLEM	3.004	0.86	117.7	173.2
HERMAS	2.954	0.84	131.4	166.0	SOKLOW	3.006	0.86	121.2	174.4
JUZRIH	2.826	0.81	123.9	176.1		3.008	0.86	119.4	177.0
	2.915	0.83	118.2	171.0		3.040	0.87	117.7	175.5
JUZRON	2.979	0.85	116.4	163.8	SOKWUN	2.967	0.85	116.8	176.5
	2.920	0.83	127.0	174.5	SOKXAU	3.003	0.86	120.1	176.2
JUZRON01	2.776	0.79	148.0	174.2	SOKXEY	3.025	0.86	117.6	177.4
JUZRUT	2.727	0.78	152.1	175.7	ULOKIP	2.823	0.81	118.5	176.9
JUZSAA	2.847	0.81	130.4	175.7		2.809	0.80	146.2	175.7
KAQSUS	2.602	0.74	131.3	177.8	ULOKIP01	2.814	0.80	118.4	177.0
LEJBUA	2.766	0.79	136.5	172.4		2.801	0.80	146.0	175.5
	3.304	0.94	140.4	160.3	VOJNAM	2.581	0.74	122.5	171.4
	2.856	0.82	142.3	175.6	WAMBOE	3.324	0.95	110.9	166.8
	2.759	0.79	132.8	176.6	WAPBOH	2.707	0.77	143.5	172.8
LICBEG	2.768	0.79	122.2	171.9	WAPBUN	2.716	0.78	128.1	172.8
LIFTOM	2.736	0.78	154.6	177.5	WAPCAU	2.720	0.78	144.6	171.5

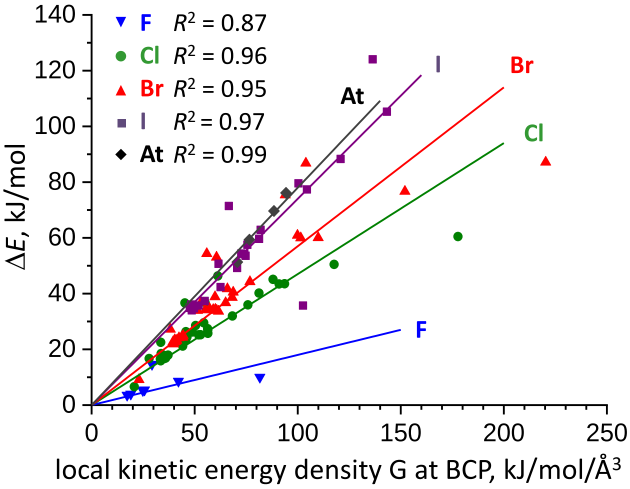

Figure S3. Correlation between calculated complexation energy ΔE and the value of local kinetic energy density G at halogen bond critical point (3;–1) for $Me_3P{=}O\cdots X–R$ halogen-bonded complexes studied in this work (X = F, Cl, Br, I, At). The solid lines correspond to one of Eqs. (9) in the main text.

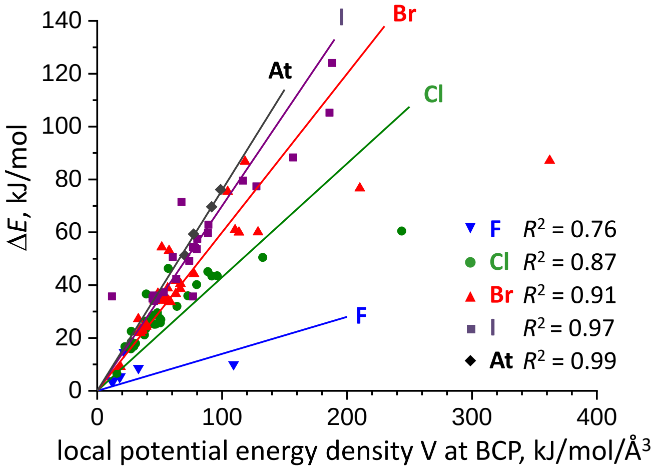

Figure S4. Correlation between calculated complexation energy ΔE and the value of local potential energy density V at halogen bond critical point (3;–1) for $Me_3P{=}O\cdots X–R$ complexes studied in this work (X = F, Cl, Br, I, At). The solid lines correspond to one of Eqs. (9) in the main text.

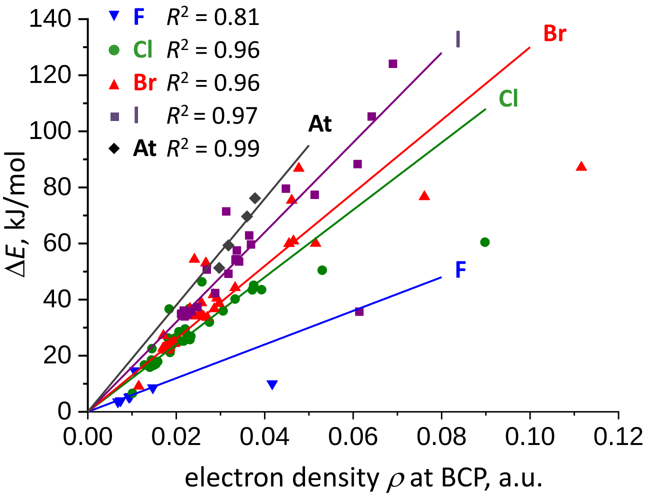

Figure S5. Correlation between calculated complexation energy Δ*E* and the value of electron density ρ at halogen bond critical point (3;–1) for $Me_3P{=}O\cdots X{-}R$ complexes studied in this work (X = F, Cl, Br, I, At). The solid lines correspond to one of Eqs. (9) in the main text.

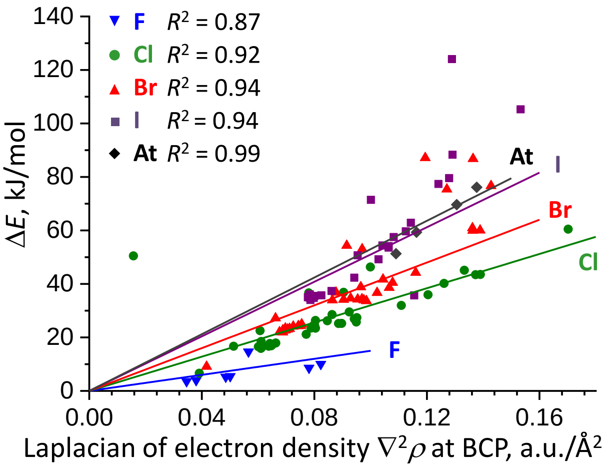

Figure S6. Correlation between calculated complexation energy Δ*E* and the value of the Laplacian of electron density $\nabla^2\rho$ at halogen bond critical point (3;–1) for $Me_3P{=}O\cdots X{-}R$ complexes studied in this work (X = F, Cl, Br, I, At). The solid lines correspond to one of Eqs. (9) in the main text.
